# Spectral graph convolutional neural network for Alzheimer's disease diagnosis and multi-disease categorization from functional brain changes in magnetic resonance images

**DOI:** 10.3389/fninf.2024.1495571

**Published:** 2024-10-30

**Authors:** Hadeel Alharbi, Roben A. Juanatas, Abdullah Al Hejaili, Se-jung Lim

**Affiliations:** ^1^College of Computer Science and Engineering, University of Hail, Ha'il, Saudi Arabia; ^2^College of Computing and Information Technologies, National University, Manila, Philippines; ^3^Faculty of Computers and Information Technology, Computer Science Department, University of Tabuk, Tabuk, Saudi Arabia; ^4^School of Electrical and Computer Engineering, Yeosu Campus, Chonnam National University, Gwangju, Republic of Korea

**Keywords:** Alzheimer's disease (AD), image classification, Convolutional Neural Networks (CNN), SGCNN model, deep learning, ablation study

## Abstract

Alzheimer's disease (AD) is a progressive neurological disorder characterized by the gradual deterioration of cognitive functions, leading to dementia and significantly impacting the quality of life for millions of people worldwide. Early and accurate diagnosis is crucial for the effective management and treatment of this debilitating condition. This study introduces a novel framework based on Spectral Graph Convolutional Neural Networks (SGCNN) for diagnosing AD and categorizing multiple diseases through the analysis of functional changes in brain structures captured via magnetic resonance imaging (MRI). To assess the effectiveness of our approach, we systematically analyze structural modifications to the SGCNN model through comprehensive ablation studies. The performance of various Convolutional Neural Networks (CNNs) is also evaluated, including SGCNN variants, Base CNN, Lean CNN, and Deep CNN. We begin with the original SGCNN model, which serves as our baseline and achieves a commendable classification accuracy of 93%. In our investigation, we perform two distinct ablation studies on the SGCNN model to examine how specific structural changes impact its performance. The results reveal that Ablation Model 1 significantly enhances accuracy, achieving an impressive 95%, while Ablation Model 2 maintains the baseline accuracy of 93%. Additionally, the Base CNN model demonstrates strong performance with a classification accuracy of 93%, whereas both the Lean CNN and Deep CNN models achieve 94% accuracy, indicating their competitive capabilities. To validate the models' effectiveness, we utilize multiple evaluation metrics, including accuracy, precision, recall, and F1-score, ensuring a thorough assessment of their performance. Our findings underscore that Ablation Model 1 (SGCNN Model 1) delivers the highest predictive accuracy among the tested models, highlighting its potential as a robust approach for Alzheimer's image classification. Ultimately, this research aims to facilitate early diagnosis and treatment of AD, contributing to improved patient outcomes and advancing the field of neurodegenerative disease diagnosis.

## 1 Introduction

Neurodegenerative disease, such as Alzheimer's disease (AD), is the most prevalent type of dementia that affects 60% to 80% of patients in the world (Turer and Sanlier, 2024; Vejandla et al., 2024). It is characterized by a decline in cognitive processes, including language, reasoning, and memory, ultimately leading to the inability to perform daily activities. The elderly population has a higher prevalence of the disease and is the fourth leading cause of death (Self and Holtzman, 2023). There is currently no cure for AD, despite extensive research and presently available medications for AD solely working on managing symptoms of the disease and putting into effect a vast financial burden for the health care system, patients and their families (Vejandla et al., 2024).

AD is progressively emerging as the most prevalent neurological disorder, with its numbers likely to rise by 2050 globally from 50 to 100 million (Zhao et al., 2024). There is an urgent need for reliable and efficient methods to detect AD at its initial stages. Early diagnosis leads to timely therapeutic interventions that potentially slow the disease progression and relieve the great burden on healthcare systems (Garg et al., 2023). Cognitive decline in AD starts many years before it is manifested clinically; the first stage may be Mild Cognitive Impairment (MCI), which may lead to AD. About 15%–20% of individuals over 60 years suffer from MCI, with 30%–35% progressing to AD within four years (Karran and De Strooper, 2022). The accumulation of coagulated tau proteins and amyloid-beta (Aβ) plaques causes neuronal death and brain shrinkage, which is the cause of the disease. This tissue loss occurs starting with the Gray Matter (GM), then going into the White Matter (WM), Corpus Callosum (CC), and extending to the Hippocampus (HC), greatly impairing neural functions (Knopman et al., 2021). Early diagnosis of AD is critical for maintaining good disease management and improving patient quality of life (Begum and Selvaraj, 2024). Modern diagnostic methods, such as PET and MRI scans, are crucial for the diagnosis of AD because they identify both structural and functional changes in the brain (Porsteinsson et al., 2021). The important information regarding the disease development from normal cognitive (NC) function through MCI to full-blown AD is provided by these imaging modalities in addition to other clinical data (Shukla et al., 2023).

New developments in machine learning technologies, especially techniques of deep learning like CNNs, have shown enormous potential in the early diagnosis and classification of AD (Wen et al., 2020). CNNs are superior in pattern recognition and image classification, which makes them ideal for large dataset image analysis, in this case, medical imaging data. Taking advantage of MRI, PET scans, and Diffusion Tensor Imaging (DTI) information, CNNs can help in the efficient and effective identification of AD and predict its progression from MCI to AD (Logan et al., 2021).

### 1.1 Research contribution

This study offers three key contributions to the field of AD diagnosis and multi-disease classification:

- We propose a novel framework based on Spectral Graph Convolutional Neural Networks (SGCNN) for diagnosing AD and categorizing multiple diseases by analyzing functional brain changes observed in Magnetic Resonance Images (MRI). Structural modifications to the SGCNN model are rigorously analyzed through ablation studies, and the performance of various Convolutional Neural Network (CNN) models, including SGCNN variants, Base CNN, Lean CNN, and Deep CNN, is systematically evaluated.
- Our study improves the reliability of AD classification tasks by implementing essential preprocessing steps such as image visualization, pixel value normalization, and precise dataset splitting. These processes ensure higher quality and consistency within the dataset, which directly enhances the performance and accuracy of the CNN models.
- Through extensive experimentation, the Ablation of SGCNN Model 1 achieves a classification accuracy of 95%, highlighting its superior potential for early detection and diagnosis of AD. This result demonstrates the effectiveness of the proposed model modifications in advancing the field of neurodegenerative disease diagnosis.

### 1.2 Research organization

This document is formatted as follows: In Section 2, the background information and relevant works are provided. Section 3 presents the proposed deep-learning approach for categorizing images associated with AD. We assess the performance of our technique and compare it with the baseline methods in Section 4. The article is concluded in Section 5, which offers suggestions for further reading.

## 2 Literature review

In the literature review, we address machine learning (ML) and deep learning (DL) methodologies for AD prediction. This section explores how these advanced methodologies contribute to improving diagnostic accuracy and understanding AD progression. Table 1 provided the overview of studies on AD prediction.

**Table 1:** Overview of studies on AD classification.

**References**	**Approach**	**Dataset**	**Classes and descriptions**	**No. of images**	**Key findings**
Rao et al. (2024)	3D convolutional neural networks with transfer learning	MRI brain images	AD, Mild Cognitive Impairment (MCI), Normal Control (NC)	1,686 images	ResNet50V2 achieved 92.15% training accuracy and 91.25% testing accuracy
Tripathi and Kumar (2024)	Speech-based cognitive impairment assessment using ML	DementiaBank's Pitt Corpus	Six classes based on cognitive impairment levels	292 recordings	Achieved 75.59% accuracy in six-class classification; XGBoost showed significant accuracy differences
Krishna et al. (2024)	DL with SMOTE data augmentation for MRI data	MRI datasets	AD, MCI, NC	2,453 images	Improved model accuracy and validity, effective for imbalanced data
Srividhya et al. (2024)	CNN-based multi-class classification	ADNI2 (sMRI)	AD, MCI, NC	1120 images	ResNet-50v2 achieved 91.84% mean accuracy, F1-score of 0.97 for AD class
Goenka and Tiwari (2023)	Multimodal DL for Alzheimer's classification	ADNI (T1-weighted MRI, AV-45 PET)	AD, MCI, NC	2,391 images	3D-Subject method achieved 93.01% accuracy, surpassing Patch-based (89.55%) and Slice-based (89.37%)
Francis and Pandian (2023)	Ensemble of pre-trained models for multi-class classification	ADNI (T1-weighted sMRI)	AD, MCI, NC	2,156 images	Achieved 85% accuracy in multi-class classification; outperformed other state-of-the-art methods
Venkatasubramanian et al. (2023)	MTDL for segmentation and classification	ADNI (structural MRI)	AD, MCI, NC	2128 images	Achieved 97.1% accuracy, 93.5% Dice coefficient, 96% accuracy for binary, 93% for multi-class classification

Biswas and Gini (2024) suggested an output-based multi-class categorization system ranging from Normal to Severe facilitates the early identification of AD. It starts by extracting hippocampal, gray and white matter from 3D MRI images and computing the volumes of each from the images using Analyze Direct and ITK Snap. Such volumes, besides other characteristics like age, gender and MMSE scores, are used to feed machine learning algorithms such as random forest, gradient boost, decision tree and KNN for the detection of Alzheimer's and the classification of the severity level of Alzheimer's. Additionally, the collected traits are arbitrarily mixed in every feasible way, including feature-level fusion, and further analyzed. The methodology is tested on two datasets, OASIS and ADNI, which were introduced in earlier sections. In the OASIS dataset, a 99% accuracy is achieved by random forest when using only white matter volume and 98% when all three volumes are integrated. For the ADNI data set, for white matter volume, the accuracy was found to be 92% for gradient boost, and for the combination of all three volumes when fused, the accuracy was 91% for both databases.

Rao et al. (2024) deal with AD by creating a new deep-learning approach that generalizes convolution networks in the third dimension to model spatial characteristics of the 3D MRI scans. The proposed classification system also uses attributes that are taken from the 3D convolutional network's several layers; however, it gives distinction importance to each layer. Using brain MRI scans from three classes (Mild Cognitive Impairment, Normal Control, AD and probability controls), the system combines transfer learning with fine-tuning. In regards to AD classification, the researchers also tried using pre-trained deep learning models such as ResNet50V2 and InceptionResNetV2, of which ResNet50V2 performed better. According to their results, ResNet50V2 achieved a testing accuracy of 91.25% and a training accuracy of 92.15%. The authors observed that the effective detection of AD utilizing 3D MRI brain images can be achieved using deep learning, particularly transfer learning with ResNet50V2.

Tripathi and Kumar (2024) suggest a method for the speech-based assessment of six kinds of cognitive impairment. After pre-processing the speech data from DementiaBank's Pitt Corpus to extract pertinent acoustic features, they train five machine learning algorithms (KNN, DT, SVM, XGBoost, and RF). Consequently, the work's output demonstrates a 75.59% accuracy rate in the six-class classification task. Besides, the significance of differences in the accuracy of XGBoost as compared to the other algorithms except the random forest is proved by the statistical tests. This approach has the potential to be used as cost- and time-effective compared to a provision of a medical diagnosis that is easily accessible in the early phases of the disease. Krishna et al. (2024) present a method that combines DL methodologies, including Deep Learning (DL), with methods of data augmentation of the SMOTE type for any MRI dataset to enhance the detection of Alzheimer's disorder. They are being used here because this approach can enhance the accuracy as well as the validity of the classification model due to the great management of the problems associated with the imbalanced data. Based on the present interdisciplinary analysis, the integration of DL with SMOTE improves the model's ability to identify AD, and this improvement was also observed when expanding its application to other forms of neurodegenerative diseases.

Srividhya et al. (2024) put forward a framework for the clustering of the stages of AD based on the AD Neuroimaging Initiative (ADNI2—Structural Magnetic Resonance Imaging—sMRI) image database. The approach entails the use of deep learning techniques, especially CNN, for a multi-class classification of AD MRI images. The emphasis is placed on choosing the most suitable pre-trained model that will be able to provide the best prediction for the AD stage of a particular patient. ResNet-50v2 was the overall best model, with a mean 91.84% accuracy and an F1-score value of 0.97 for the AD class. They used Grad-CAM and Saliency Map to visualize the highest accuracy model to know which part of the image the algorithm concentrated on for classification. Kaya and Çetin-Kaya (2024) put forward a framework that involves the use of PSO to adjust the hyperparameters of CNN for the detection of AD from MRI data. The approach comes in handy to fine-tweak hyperparameters like a number of convolution layers and filters and other issues like lack of labeled data, high inter-class similarity, and overfitting. As for the proposed lightweight model, it attains a test accuracy of 99.53%, and an F1-score of 99.63% of the tests were performed on a public dataset, which was higher than those obtained in prior studies and could be highly useful to help clinicians in the diagnosis and decision-making process.

By utilizing MRI data from the ADNI dataset, the El-Assy et al. (2024) provide a cutting-edge CNN design for the classification of AD. The two CNN models used by the network have varying filter widths and pooling layers. Nonetheless, these two models are combined for classification purposes because the system handles three, four, and five categories. With 99.43%, 99.57%, and 99.13% accuracy, respectively, the suggested CNN architecture produces comparatively high results. These results demonstrate the suggested network's ability to extract features from MRI scans and differentiate between various AD subtypes and stages, assisting medical professionals in accurately and promptly diagnosing AD patients. Khan et al. (2024) introduce their new multimodal fusion-based approach called Dual-3DM3-AD to diagnose AD from the MRI and PET image scans accurately and in the early stages. The management starts with the pre-processing of both image types: For the noise reduction of the raw data, a Quaternion Non-local Means Denoising Algorithm (QNLM) is applied. Subsequently, the Morphology function is used for skull stripping, resulting in an improved image quality further refined with the help of a Block Divider Model (BDM) to convert the 2D image into a 3D image. The model incorporates semantic segmentation using a Mixed-transformer with Furthered U-Net with Complexity Minimization. It employs the Densely Connected Feature Aggregator Module (DCFAM) for feature aggregation and implements a multi-scale feature extraction to extract features from the segmented images it obtains. There is then feature dimensionality reduction by applying multi-head attention, wherein a softmax layer is used, covering multi-class diagnosis of Alzheimer's. The proposed Dual-3DM3-AD achieves a high accuracy of 98% and a high sensitivity of 97.8%, specificity of 97.5%, F-measure of 98.2%, and ROC curves that are statistically significantly better than any other existing model for multi-class Alzheimer diagnosis.

Hu et al. (2024) study leverage Graph Neural Networks (GNNs) with claim data to predict AD and Related Dementia (ADRD) risk. A variational GNN (VGNN) with a relation importance method was used to estimate ADRD likelihood and provide explanations of feature importance. Three prediction scenarios (1-, 2-, and 3-year windows) were analyzed, and VGNN performance was compared to the Random Forest (RF) and Light Gradient Boost Machine (LGBM) models. Across all scenarios, the VGNN outperformed RF and LGBM models, with AUROC improvements of over 9%–10%. The VGNN showed strong predictive ability, with AUROC scores ranging from 0.7001 to 0.7480, highlighting its efficacy in ADRD risk prediction. In Amini et al. (2024), Natural Language Processing (NLP) techniques combined with machine learning methods were utilized to develop an automated approach for predicting the progression from MCI to AD within a 6-year timeframe based on speech data. The study analyzed neuropsychological test interviews of 166 participants from the Framingham Heart Study, comprising 90 cases of progressive MCI and 76 cases of stable MCI. The best-performing models incorporated speech-derived features along with demographic factors such as age, sex, and education level, achieving an accuracy of 78.5% and a sensitivity of 81.1% in predicting MCI-to-AD progression.

Goenka and Tiwari (2023) use T1-weighted MRI and AV-45 PET images from the ADNI database to provide a unique multimodal deep-learning model for the categorization of AD. They use three cutting-edge approaches: 3D-Subject, 3D-Patches, and 3D-Slices. The 3D-Patches, a unique feature, include patches of different sizes from 32 to 88 for feature extractions. In contrast, the 3D-Slices, another novel approach, include uniform slicing interpolation zoom and subset slicing to generate slices from 8 to 64. With the aid of the Ensembled Volumetric ConvNet, the model achieves an impressive accuracy of 93.01% for AD vs. NC vs. MCI. Notably, the 3D-subject-based method, a pioneering approach, yields the highest accuracy, 93.01%, surpassing the Patch-based (89.55%) and Slice-based (89.37%) methods. Using T1-weighted structural MRI images of the brain from the AlzhAlzheimer'sease Neuroimaging Initiative database, the authors in Francis and Pandian (2023) present an algorithm that integrates the last layers of pre-trained models Xception, Inception V3 and MobileNet for the AD and related cognitive states classification. The algorithm is tested with a multi-class classification problem, and the accuracy obtained is about 85%. It provides specific accuracies of 85% for distinguishing Mild Cognitive Impairment convertible (MCI) from Mild Cognitive Impairment non-convertible (MCInc), 94% for classifying AD from cognitively normal (CN), and 92% for differentiating MCIc from CN. The results demonstrate that the proposed algorithm surpasses other state-of-the-art methods in multi-class classification and in differentiating MCIc from MCInc.

Adaobi et al. (2023) employed a fine hybrid of Xception and Fractalnet-based deep learning techniques for the classification of the phases of AD into five stages. MRI images were drawn from the ADNI dataset to enhance the performance of the model, and an attempt was made to utilize appropriate pre-processing techniques together with segmentation procedures based on Unet++ algorithms. Recall, precision, and accuracy are established as the evaluation metrics of the performance of the proposed approach. These results of the investigation indicate that the proposed technique can achieve a level of accuracy of 98.30% recall, 99.72% precision and 99.6% accuracy in multi-class classification. To summarize, the findings point to the fact that the presented methods, when integrated with MRI images, can be useful in the classification and prediction of neurodegenerative diseases, such as AD. Venkatasubramanian et al. (2023) trained a deep learning model for the segmentation and automatic categorization of AD using structural MRI data. They adopted MTDL for the joint segmentation of the hippocampus in the given images, a comprehensive approach. The deer hunting optimization (DHO) is then used to fine-tune the CNN model (capsule network) for the categorization of disease, guaranteeing a strong and trustworthy classification procedure. The typical method has been applied to ADNI-standardized MRI datasets, and it is effective, as suggested above. It is discovered that the proposed MTDL achieves 97.1% accuracy and 93.5% of the Dice coefficient. In comparison, the suggested MTDL model achieved a 96% accuracy for binary classification and a 93% accuracy for multi-class classification. These thorough evaluation results instill confidence in the validity and reliability of the proposed technique.

## 3 Proposed framework

**Figure 1:**
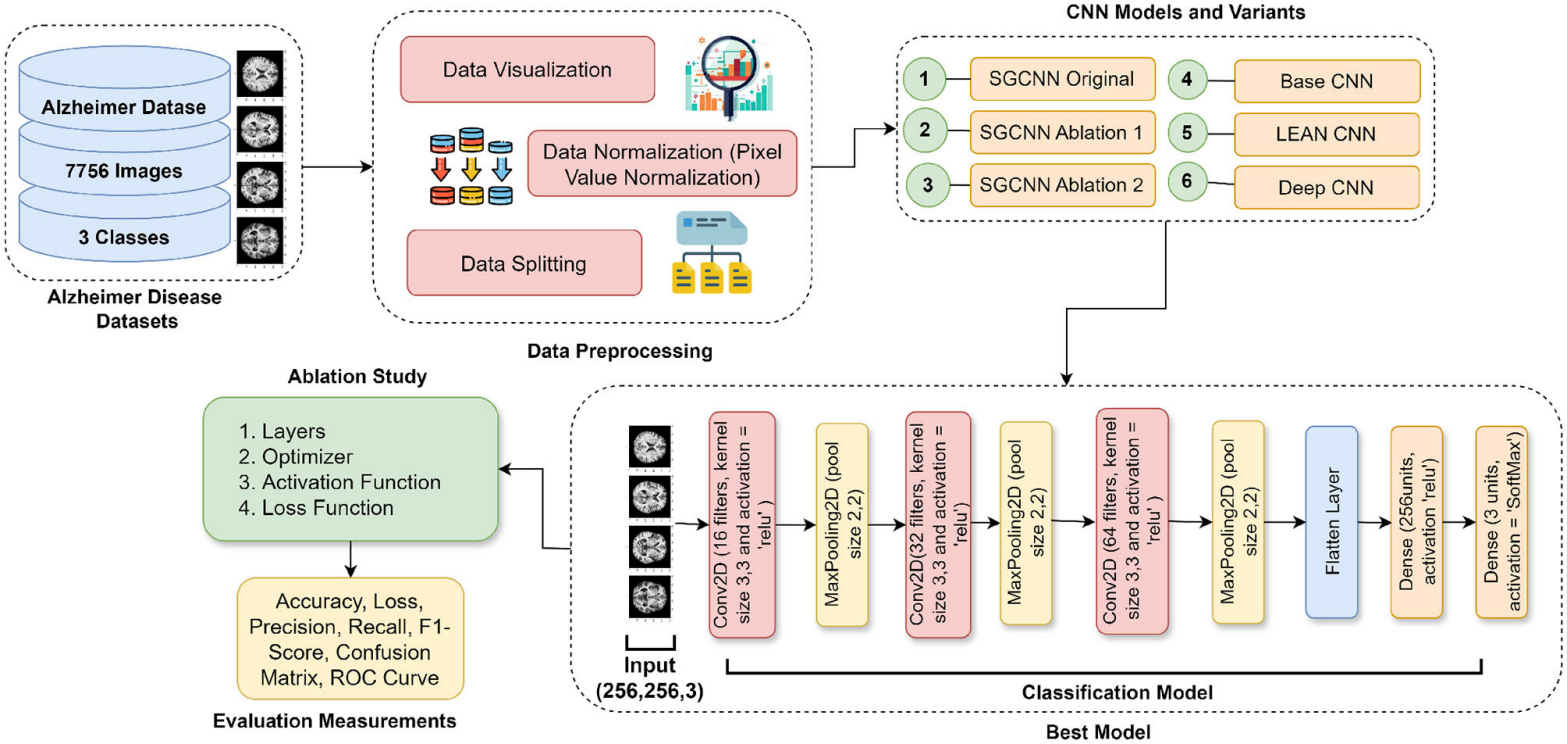
Proposed framework for Alzheimer's disease detection.

The suggested methodology for utilizing deep learning models to identify AD is described in this section. In the classification of AD, Figure 1 presents a holistic view of deep learning models. The process starts with an experimental dataset of 7,756 images belonging to three categories. Data pre-processing covers visualization of data, normalization and data split. Next, the framework discusses model selection; SGCNN is compared to base CNN, Lean CNN, and deep CNN, as well as several ablation variants of SGCNN. The best-identified model is the sequential model of convolution, which is obtained by using a Conv2D layer of 16 filters followed by a series of layers of 32 and 64 filters, respectively, of a maximum pooling layer, then a flattening layer and two dense layers. Lastly, there is one final dense layer of three neurons with softmax activation to spit out the predictions for the three classes. The ablation study aims to determine the sensitivity of the model to hyperparameters, including loss function, learning rate, batch size, optimizer and activation function. In the last section, the results and analysis are presented with reference to the Receiver Operating Characteristic (ROC) curve, accuracy, loss, precision, recall, F1 score, confusion matrix, and accuracy. This elaborate work is meant to ensure the best deep learning model and hyperparameters that enable accurate classification of Alzheimer's disorders.

### 3.1 Experimental dataset

This paper focuses on categorizing participants into three groups: Alzheimer's disease (AD), Parkinson's disease (PD), and CONTROL. As a reference point for comparison, CONTROL stands for healthy people free of neurological conditions. Subjects with AD, a neurological illness depicted by a decline in cognition and memory, are included in the class. The participants in the PD class have been diagnosed with Parkinson's disease, which is typified by stiffness and tremors in the muscles. Data from clinical examinations, medical imaging, and other modalities that represent the neurological and physiological aspects of these illnesses are probably included in the dataset.

Two directories—training images and testing images—are included in the collection. In this study, we make a new directory to hold the combined photographs.

### 3.2 Data pre-processing

Preprocessing data is crucial for deep learning and data assessment systems. The data needs to be cleaned and altered to prepare it for additional analysis or training of deep learning models. For data preprocessing, this study used data visualization and normalization, both of which enhanced the effectiveness of the suggested approaches.

**Figure 2:**
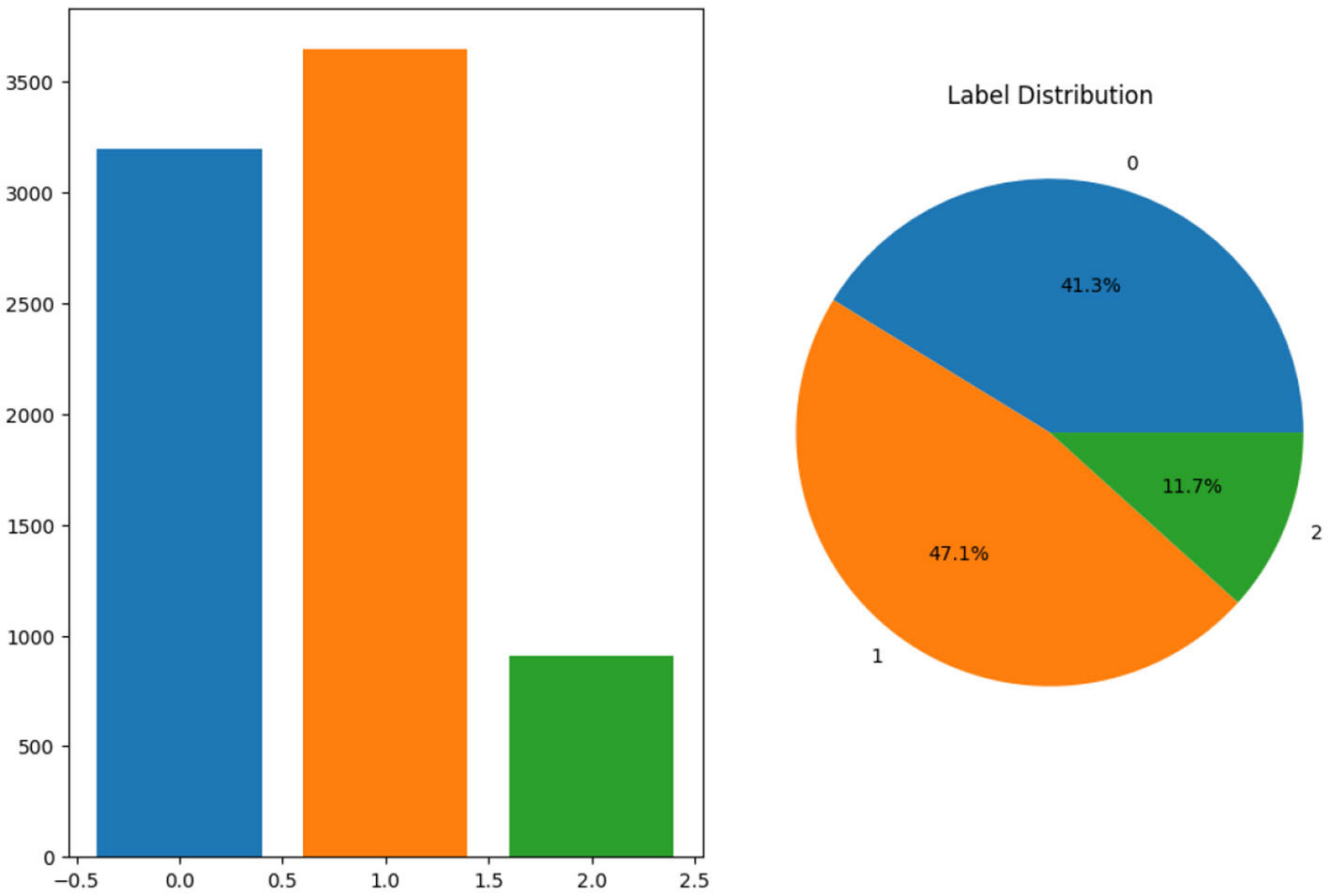
Label distribution.

**Figure 3:**
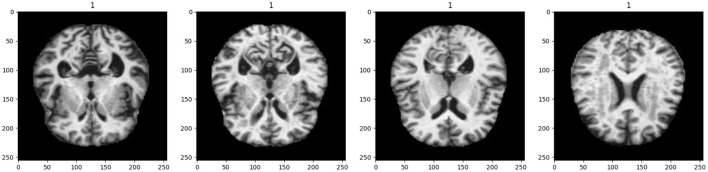
Sample MRI images.

Figure 2 shows the proportion of each label in the pie chart and a bar chart to demonstrate the distribution of labels. The bar chart on the left displays the frequency of each label, showing that label 1 has the highest count, with ~3,500 samples, followed by label 0, with around 3,000 samples, and label 2, which has the smallest count of roughly 1,000 samples. On the right, the pie chart provides a proportional breakdown of the dataset. It shows that 41.3% of the data belongs to label 0, 47.1% to label 1, and the remaining 11.7% to label 2. Overall, label 1 dominates the dataset, while label 2 represents the least frequent category. Images from the dataset batch are shown in Figure 3, which gives a visual representation of the dataset.

An essential first step in getting data ready for deep learning models is normalization. A normalized function defined in the script accepts a picture *x* as input and its label *y*. The maximum pixel value in the image is determined by *x*_*max*_. Function. By dividing the image by *x*_*max*_, it normalizes it and guarantees that the values of pixels are scaled within the range of 0 and 1. Enhancing convergence rates and averting problems like gradient vanishing aids in the stabilization of the training process. After using the map method to apply this normalized function to the original dataset, the normalized dataset is created and saved in the variable data, ready to be fed into the model. Original Before normalization, the data range in the batch was from 0.0 to 254.42578. This represents the original pixel intensity values in the image data, where the maximum pixel value was close to 255, typical for 8-bit grayscale images. After applying the normalization function, the data range was scaled between 0.0 and 1.0. This was done by dividing each value of the pixel by the highest value in the batch, effectively normalizing the image data to a common scale suitable for neural network input. Data is separated into testing, validation, and training sets following data normalization to make sure the model is trained, validated, and tested on various subsets of the data. This segment is essential for assessing the model's functionality and generalization capacity. The training set is extracted using the take technique, which takes a predetermined piece of the dataset—usually the largest portion—while the testing and validation sets are extracted using the skip approach, which removes these training samples. Typically, 80% of these sets are used for training, 10% are used for validation, and 10% are used for testing. Now, the training size is 194, and the Validation size and test size are 24. Table 2 provides the comparison of original and normalized data sizes and ranges.

**Table 2:** Comparison of original and normalized data batches: data size and range.

**Batch**	**Data size**	**Data range (min–max)**
Original batch	Varies, e.g., (batch_size, height, width)	0.0–254.42578
Normalized batch	Same as original	0.0–1.0

Algorithm 1 depicts the workflow that is followed in order to pre-process the data. The first step is the input of the dataset, which itself consists of training and testing images. It then makes a new directory for merged images and visualizes the dataset in order to see the distribution of labels and example images. By dividing each image by its greatest value, it first scales the pixel values of the image in the range of 0–1. Next, the dataset is divided into the following ratios: 80:10:10 for the training, validation, and test sets. Finally, the pre-processed dataset is presented in a form that is suitable for training a model.

**Algorithm 1:**
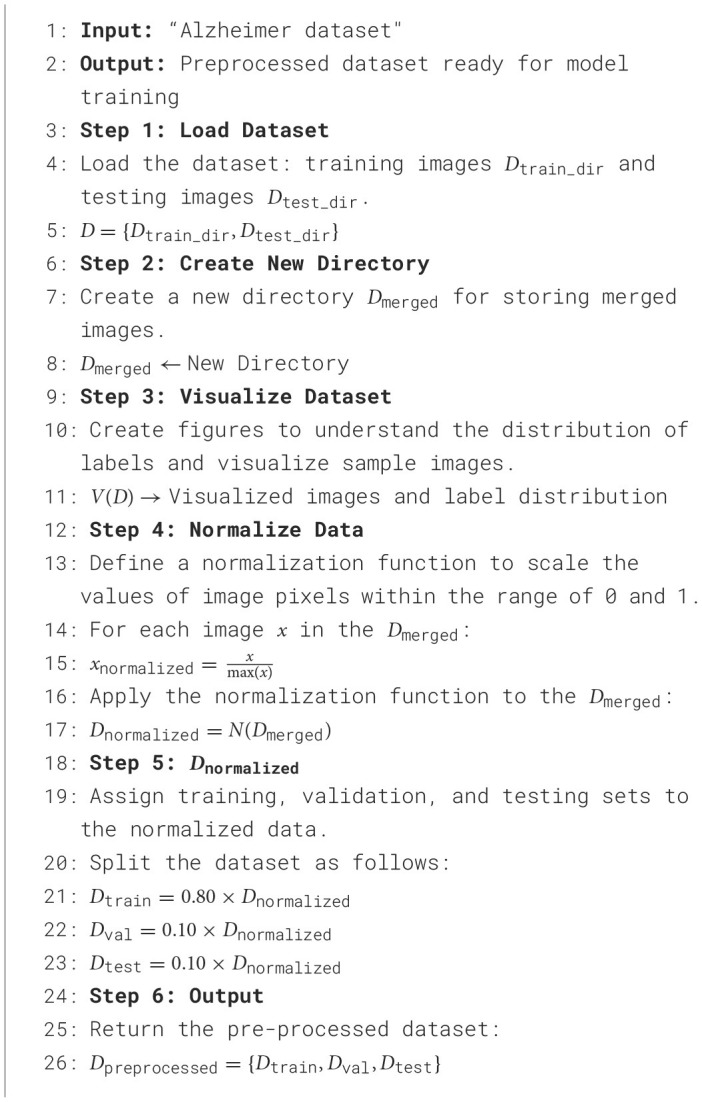
Experimental dataset and pre-processing steps.

### 3.3 Deep learning model

Deep learning models are sophisticated neural networks made to recognize and extract information from large, complex datasets automatically. These models are very effective for tasks like picture classification, audio recognition, and natural language processing because they are composed of numerous layers, each of which processes data to record increasingly abstract representations. This study conducted an ablation study on many deep learning models, including the Base CNN Model, LEAN CNN Model, Deep CNN Model, and SGCNN Original Model.

#### 3.3.1 SGCNN original model

An effective deep learning model for graph-structured data is the Spectral Graph Convolutional Neural Network (SGCNN), which can identify intricate patterns and connections in non-Euclidean structures such as molecular graphs and social networks. Unlike conventional CNNs, SGCNNs use spectral-domain convolutional processes, which makes them useful for tasks like graph and node classification. Using the best features of both architectures, the hybrid deep learning model combines a segmentation and a classification model. Utilizing a U-Net architecture, the segmentation model processes images through convolutional layers activated by ReLU after first utilizing an input layer for 256 × 256 RGB images. Max pooling is then utilized to minimize the spatial dimensions of the processed images. The last convolutional layer creates the segmentation mask, while an upsampling layer recovers the image size.

**Figure 4:**
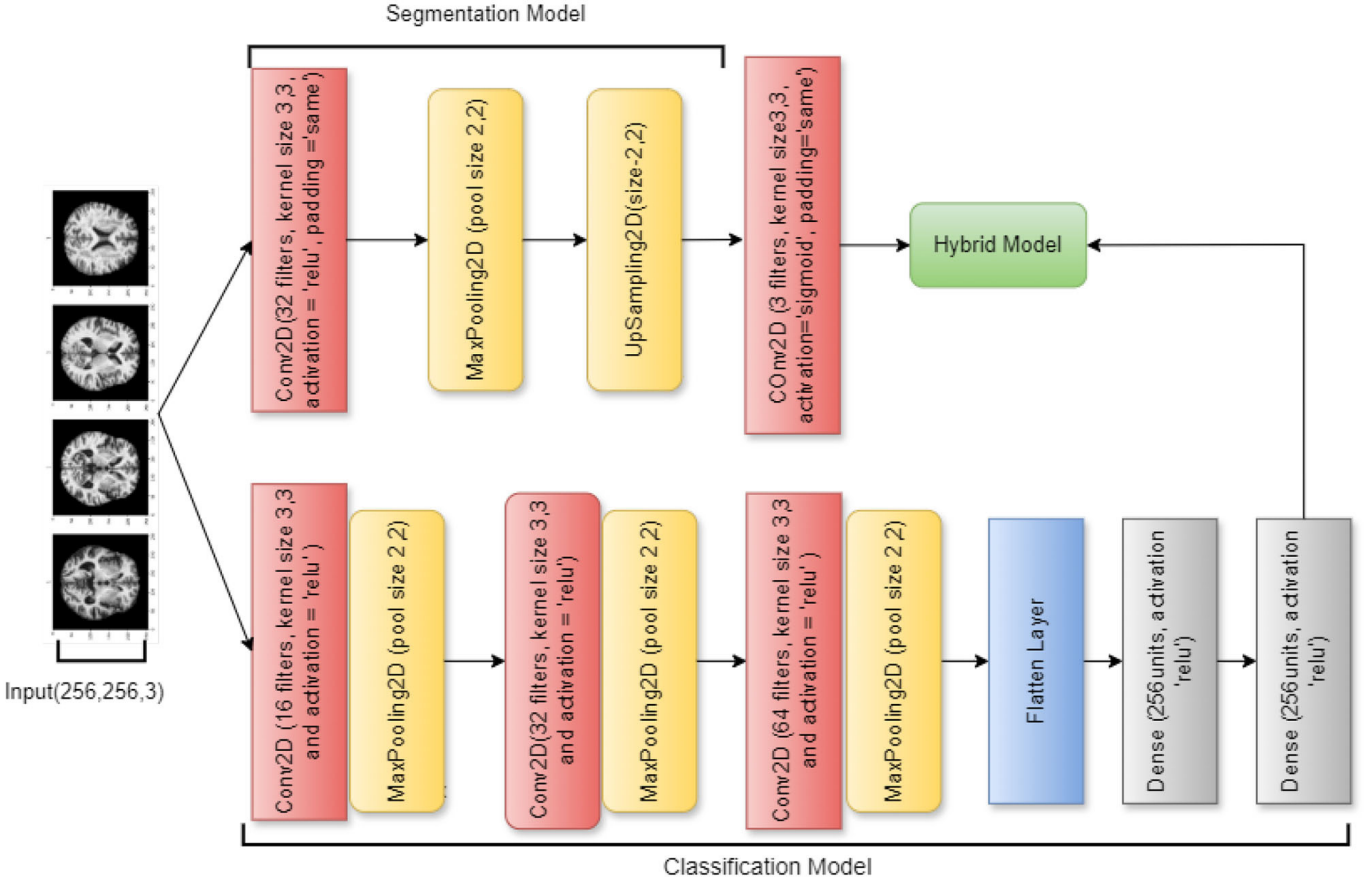
SGCNN model architecture.

A CNN is used in combination with this classification model to classify images such as PD, AD, and CONTROL. Several convolutional and max pooling layers are added after the input layer in order to extract features. The final softmax layer for classification is reached after the feature maps have been flattened and dense layers with ReLU activation have captured complex patterns. The hybrid model incorporates the classification model with the segmentation output of the U-Net network. With the use of precise spatial data, this method improves classification accuracy while enabling the model to execute segmentation and classification tasks. Overall performance in identifying the input images is improved by the combined model's excellent integration of segmentation characteristics. Figure 4 visualizes the architecture of the original SGCNN model.

#### 3.3.2 Ablation of SGCNN model 1

An ablation study of the SGCNN model is the second model, with the primary goal of focusing only on classification problems and streamlining the architecture by eliminating the segmentation component. This model simplifies the network into a more conventional CNN by removing the segmentation layers while keeping a topology resembling that of the original SGCNN. It starts with several convolutional layers and moves on to pooling layers and dense layers for classification. An input layer for 256 × 256 RGB images is the first layer in the design. Three convolutional layers with progressively larger filter sizes (16, 32, and 64) come next. To lower the spatial dimensions, a max-pooling layer is paired with each convolutional layer.

**Figure 5:**
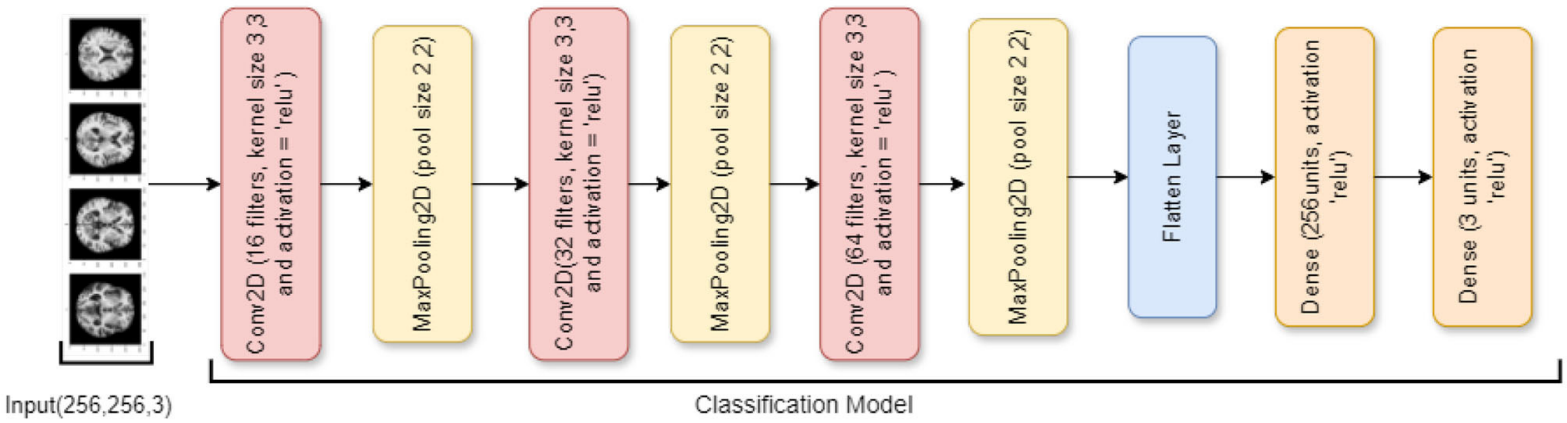
Ablation of SGCNN model 1 architecture.

The elimination of the segmentation network, which was a feature of the SGCNN, is the most notable modification to this model. Because of the network's amplification brought forth by this ablation, the performance of the classification component can be examined more closely. The model is simpler now that the segmentation layers have been eliminated, and it only concentrates on classifying the input images into three groups (e.g., CONTROL, AD, and PD). Like the original model, the model is compiled using the Adam optimizer with category cross-entropy loss. It is trained with a batch size of 32 across 15 epochs. Figure 5 visualizes the architecture of the ablation of the SGCNN model 1.

#### 3.3.3 Ablation of SGCNN model 2

The architecture of this third model, which is an additional ablation study of the SGCNN model, is largely unchanged from the earlier iterations. However, there are a few significant changes. The model starts with a modified segmentation model that has an upsampling layer to boost spatial dimensions and a 32 filters-convolutional layer. In contrast to the original segmentation model, this variant generates the segmentation output in the last layer using a sigmoid activation function.

**Figure 6:**
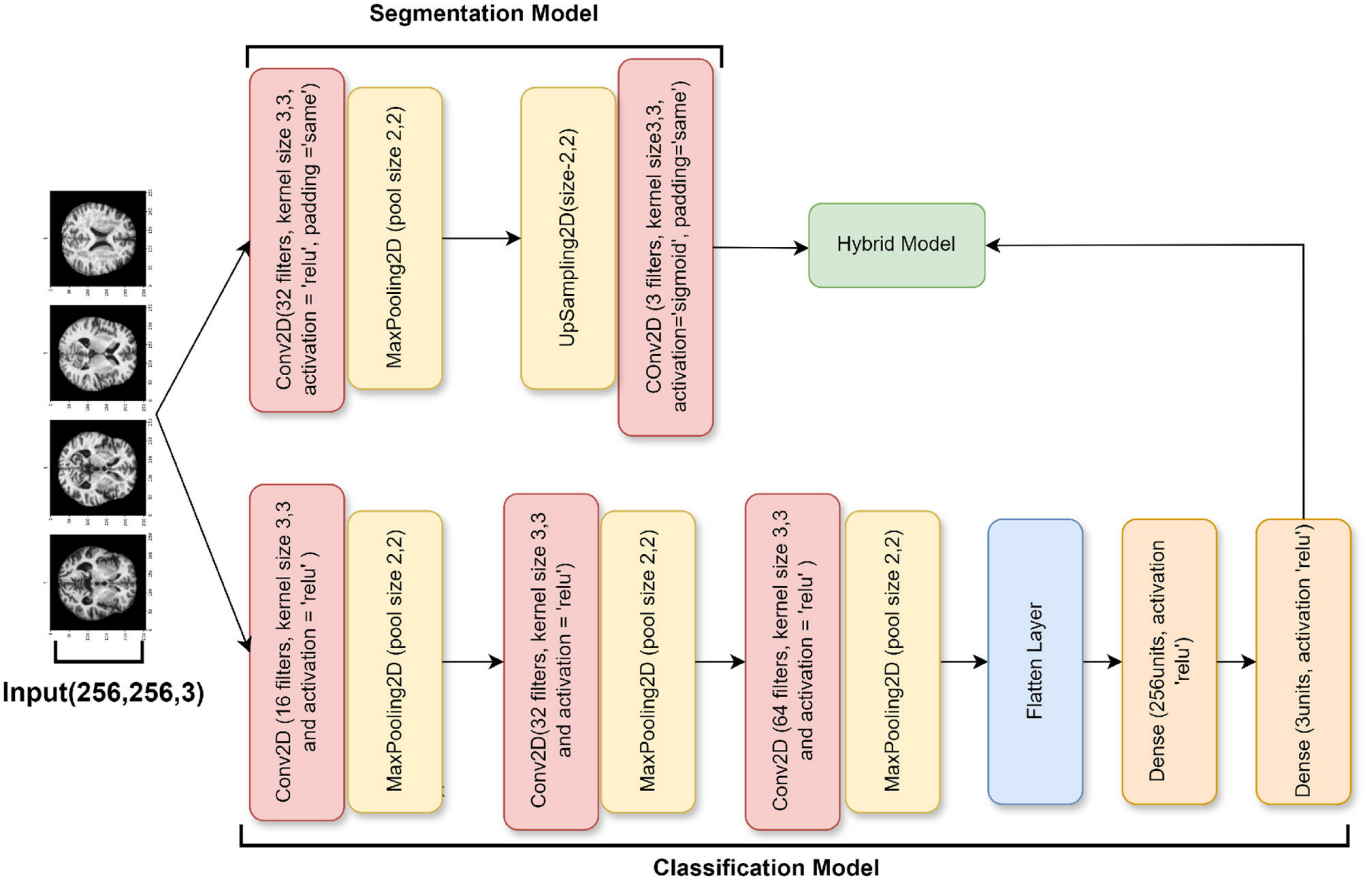
Ablation of SGCNN model 2 architecture.

In contrast to the earlier models, the classification model component adds a 64-filter convolutional layer and removes the batch normalization and dropout layers from the dense layer. With these modifications, the classification model becomes more simplified and produces the classification output with softmax activation by connecting the flattened feature maps straight to the final dense layer. The design is put together utilizing the Adam optimizer and category cross-entropy loss, the same as the earlier models. The metrics used to evaluate the model's performance are test accuracy, validation, and training. Figure 6 visualizes the architecture of the ablation of the SGCNN model 2.

#### 3.3.4 Base CNN model

The purpose of this base convolutional neural network (CNN) model is to categorize images. The design is simple, with layers processing and classifying input photos in a stack order. The model begins with a convolutional layer that applies 16 3 × 3 filters to the 256 × 256 input images, each of which has three RGB color channels. This layer introduces non-linearity using the ReLU activation function. Then, by normalizing the convolutional layer's output, batch normalization stabilizes the training process. Subsequently, the model comprises a max-pooling layer that reduces the computational complexity and concentrates on the most prominent characteristics by downsampling the feature maps' spatial dimensions by a factor of two.

**Figure 7:**
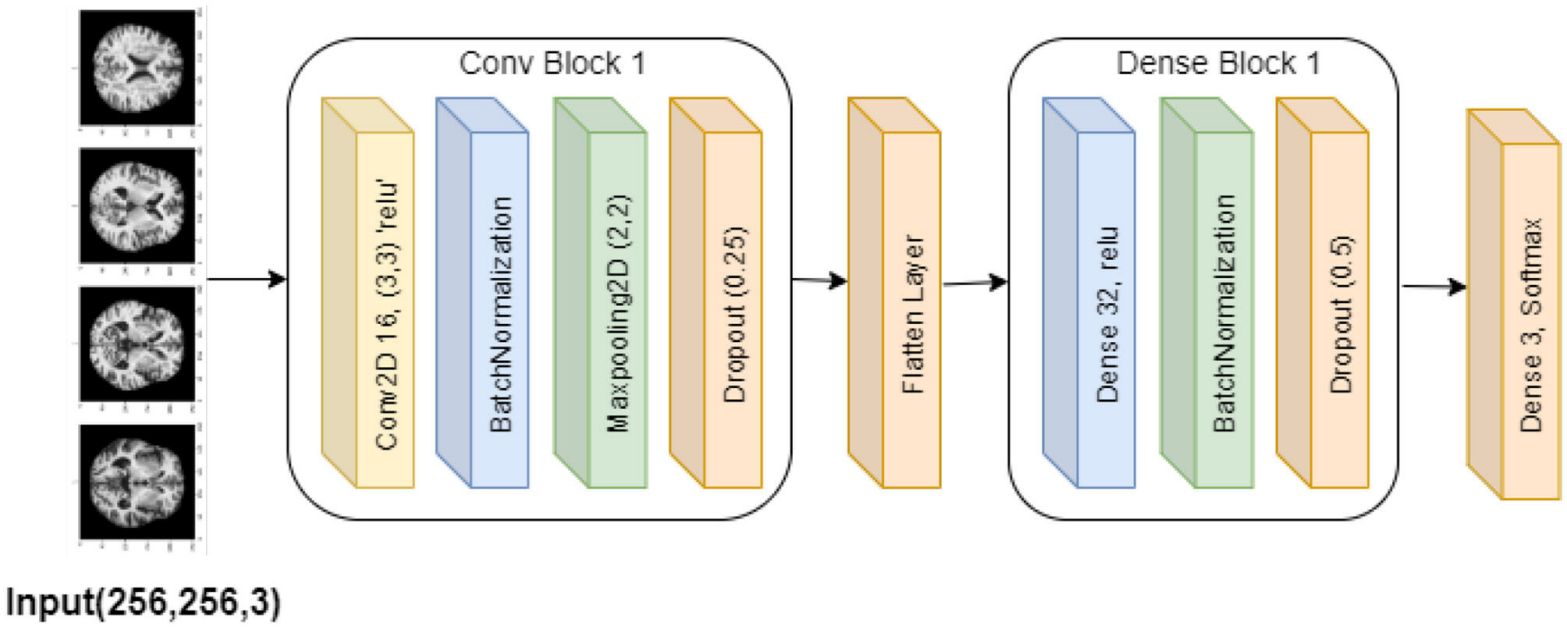
Base CNN model architecture.

The model then incorporates a dropout layer, which randomly removes 25% of the neurons during training in order to avoid overfitting. In order to prepare it for the thick layers that come next, the output is then flattened into a 1D feature vector. Using the ReLU activation function once more, the dense layers begin with a completely connected layer comprising 32 neurons. Batch normalization and an additional dropout layer—which removes 50% of the neurons—come after this layer to further lessen the possibility of overfitting. The classification output is produced by an output layer in the model's stages, which employs the softmax activation function to generate a probability distribution over three classes. The model is constructed using the standard techniques for classification tasks: the Adam optimizer and categorical cross-entropy loss. Throughout the training, the model's accuracy is monitored to make sure it picks up the picture classification skills correctly. Figure 7 visualizes the Base CNN model's architecture.

#### 3.3.5 LEAN CNN model

**Figure 8:**
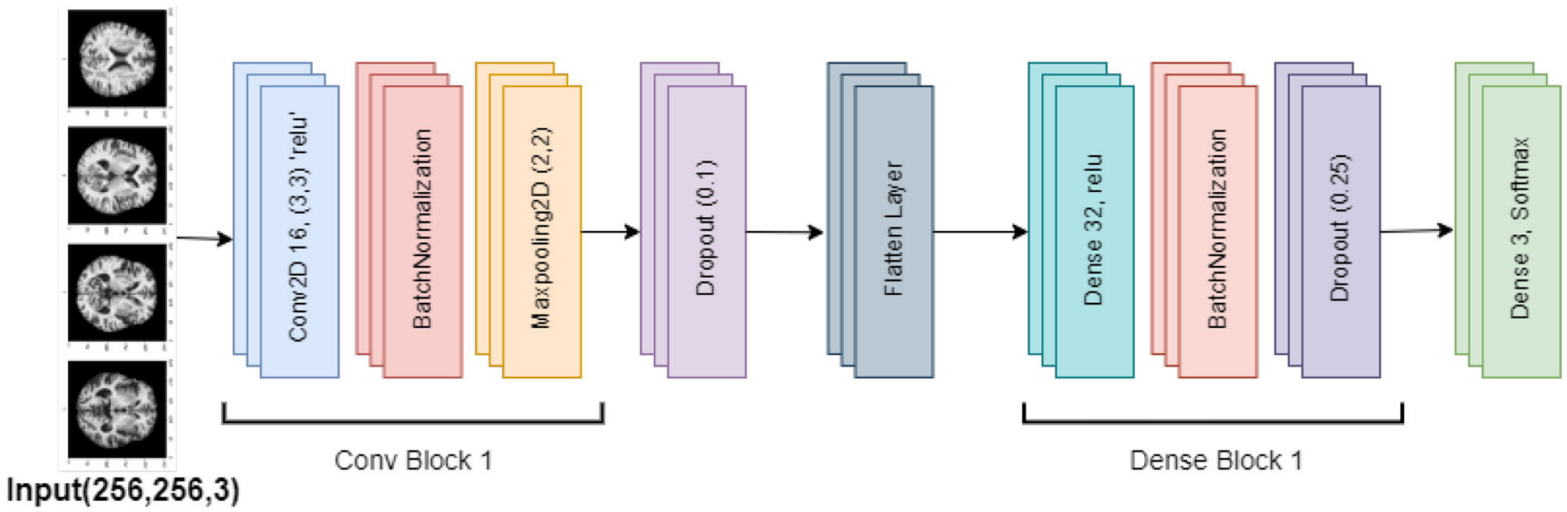
Lean CNN model architecture.

The Lean CNN model is based on the Base CNN model, which incorporates adjustments to minimize overfitting. It introduces particular adjustments to dropout rates while maintaining a similar design. This model is composed of a stack of successive layers, starting with a convolutional layer that processes 256 × 256 × 3 (RGB) input pictures using 16 filters of size 3 × 3. The convolutional layer also uses ReLU activation. Next, the training is stabilized using batch normalization and the feature map dimensions are minimized using max pooling. Lower dropout rates are the primary change made to the Lean CNN model. In particular, compared to 0.25 and 0.5 in the Base CNN model, the dropout rate is reduced to 0.1 in the first dropout layer and 0.25 in the second dropout layer. By lowering the chance of overfitting, this modification is intended to lessen the degree of regularization, which could enhance the model's performance. Figure 8 visualizes the architecture of the Lean CNN model.

The feature maps are processed via dense layers after being flattened into a 1D vector. After the 32 units of dense layer with ReLU activation, there is a 0.25 dropout rate and batch normalization. The model's final layer uses softmax activation to create a dense output layer with three neurons that produce the classification probabilities. Overall, the lower dropout rates indicate a deliberate change meant to improve model performance by reducing overfitting, even if the Lean CNN retains the Base CNN's fundamental architecture.

Algorithm 2 explains the design of some SGCNN and CNN models for image classification, which are as follows. The algorithm initializes a sequential model and defines five different models, Including SGCNN Model 1, SGCNN Model 2, SGCNN Model 3, Base CNN Model, Lean CNN Model, and Deep CNN Model. Each model has six layers of convolution, batch normalization, max pooling, dropout, density layers, and one output layer that uses softmax in the form of probabilistic distribution. One model has fewer filters and smaller kernel sizes and uses a dropout rate of 0.2. In comparison, the second model has more filters, larger kernel sizes and a dropout rate of 0.3. The algorithm also defines the base layers applicable to all models such as a flattening layer, dense layers as well as the output layers. The model is then trained using the training data and verified using the validation set. Finally, it is stated that the prior model was assembled using the Adam optimizer and categorical crossentropy loss function.

**Algorithm 2:**
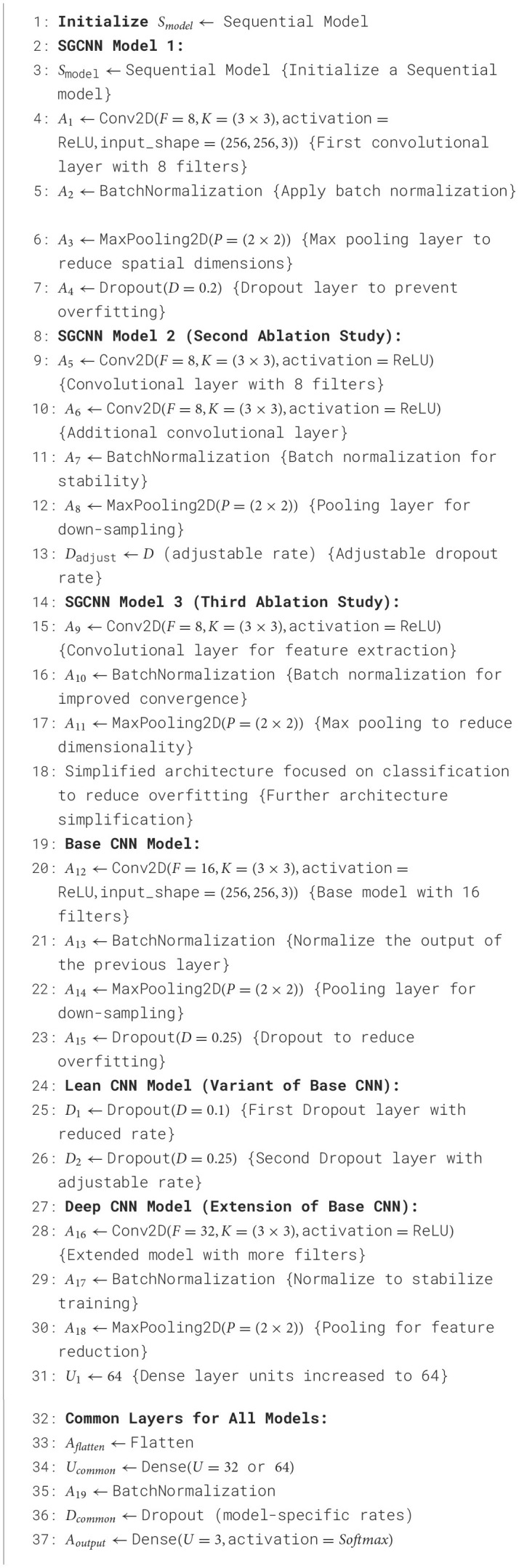
SGCNN and CNN model variants.

#### 3.3.6 Deep CNN model

The Base and Lean CNN models are built upon the Deep CNN model, which increases the architecture's complexity by using more layers and units. More convolutional layers in this model improve its capacity to extract fine-grained characteristics from the input images, which makes it more appropriate for challenging classification tasks. Two extra convolutional layers, each with 32 filters, are added to the original 16-filter convolutional layer in the Deep CNN model. These additional layers let the model recognize more complex patterns and enable it to extract deeper characteristics from the incoming data. Each convolutional layer is followed, like in the earlier models, by batch normalization and max pooling, which downsamples the feature maps.

**Figure 9:**
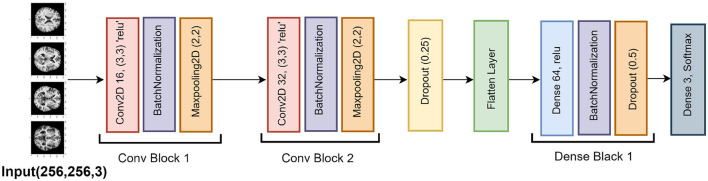
Deep CNN model architecture.

Additionally, the Base and Lean CNN models' units in the dense layer are increased to 64 units in the Deep CNN model. With more neurons, the model can process the bigger feature set generated by the further convolutional layers. With a 25% dropout after the convolutional layers and a 50% dropout after the dense layer to avoid overfitting, the dropout rates are still in line with the earlier models. Overall, the extra convolutional layers and the larger dense layer distinguish the Deep CNN model from the Base and Lean CNN models. By extracting more precise features from the data, these improvements should increase the model's accuracy in classifying photos. Figure 9 visualizes the Deep CNN model's architecture.

### 3.4 Ablation study

In this study, ablation analysis was conducted to analyze the effect of structural modifications on the performance of the SGCNN architecture for Alzheimer's image classification. The SGCNN Original Model served as the baseline, achieving a classification accuracy of 93%. Two variants of the SGCNN model were developed to assess the effects of different structural changes: Ablation of SGCNN Model 1 and Ablation of SGCNN Model 2. The first variant, Ablation of SGCNN Model 1, incorporated specific architectural adjustments that led to a notable improvement in classification accuracy, reaching 95%. This significant enhancement demonstrates the effectiveness of these modifications in refining the model's capacity to distinguish between AD stages.

Conversely, the Ablation of SGCNN Model 2 did not exhibit an improvement over the baseline, maintaining the same 93% accuracy as the original model. This finding emphasizes how crucial it is to choose suitable structural changes to achieve performance gains. The ablation study underscores the potential of targeted architectural adjustments in optimizing model accuracy for medical image classification tasks. By isolating and analyzing these modifications, the study provides valuable insights into effective strategies for enhancing diagnostic tools for AD, offering promising avenues for further investigations and advancements in this critical field of medicine. Among the models, the Ablation of SGCNN Model 1 performed the best, achieving the highest accuracy of 95%.

## 4 Experimental result and discussion

This section provides the evaluation measurements used in this study and the experimental results of all the models.

### 4.1 Evaluation measurements

The effectiveness of the suggested methodology is calculated in this study utilizing a variety of evaluation measures, including F1-score, recall, accuracy, and precision. These crucial assessment metrics offer comprehensive details regarding how the suggested technique should be interpreted. The first metric that is frequently seen as the foundation of performance evaluation is accuracy. By considering the total number of instances, the percentage of correctly detected outcomes is calculated using the accuracy metric, which is defined as the ratio of correctly predicted positive and negative cases (true positives and true negatives) to the total number of instances, including false positives and false negatives as shown in Equation 1.


(1) $$\begin{array}{l} Accuracy=\frac{TP+TN}{TP+TN+FP+FN} \end{array}$$

(2) $$\begin{array}{l} Precision=\frac{TP}{TP+FP} \end{array}$$

Precision is important in situations where the cost of false positives is high, as it measures the model's ability to avoid incorrectly predicting negative instances as positive. However, precision does not account for how many actual positive instances the model missed. Equation 2 explains the precision. Recall is especially important in scenarios where missing positive instances (false negatives) has serious consequences, such as in medical diagnoses. A high recall indicates that the model captures most of the positive instances, but it may come at the expense of higher false positives. Equation 3 defines the precision. To balance the trade-offs between precision and recall, the F1-score is used. The F1-score is the harmonic mean of precision and recall, ensuring that both are taken into account.


(3) $$\begin{array}{l} Recall=\frac{TP}{TP+FN} \end{array}$$

(4) $$\begin{array}{l} F1-score=2\times \frac{Precision+Recall}{Precision+Recall} \end{array}$$

The F1-score is particularly useful when the dataset is imbalanced and when both false positives and false negatives are important to consider. It provides a single metric that captures a balance between precision and recall, allowing for more informed model performance evaluations. Equation 4 demonstrated its computation. A classification model's performance can be categorized and assessed using a confusion matrix, which provides a list of counts for true positives (TP), true negatives (TN), false positives (FP), and false negatives (FN). It offers details about the model's capacity for learning and differentiating between classes. While genuine positives and true negatives demonstrate appropriate classifications, false positives and false negatives highlight instances in which the model misclassifies. Identifying specific types of errors the model makes and guiding modifications to enhance its performance are made possible in large part by this matrix. A binary classification model's performance at different thresholds is represented graphically by a curve known as a Receiver Operating Characteristic (ROC). True positive rate (TPR) against false positive rate (FPR) plotting displays the proportion of correctly labeled positive instances on the *y*-axis and the proportion of falsely identified positive cases on the *x*-axis. Curves further from the diagonal line indicate better model performance, which is the representation of plotting random guesses. The left-hand corner of the plot denotes improved performance and accuracy. Increases in the model's performance are indicated by higher values of the area under the curve (AUC).

### 4.2 Result and findings

Table 3 shows the classification report of several pre-trained CNNs, each one evaluated with a different configuration on a dataset. In the table, values in terms of precision, recall, F1-score, support for each class and the weighted average over all classes (Wei. Avg) are provided. The SGCNN Original Model shows strong performance, particularly in Class 1, with a precision value of 0.99 and a recall value of 0.99, resulting in an F1-score value of 0.99 with support of 355 instances. Class 2 exhibits a slightly lower performance, with a precision value of 0.95, a recall value of 0.91, and an F1-score value of 0.93, supported by 343 instances. The model struggles more with Class 3, achieving a precision value of 0.68, a recall value of 0.83, and an F1-score value of 0.75, with a support of 70 instances. The precision, recall, and F1-score weighted averages are 0.95, 0.94, and 0.94, respectively.

**Table 3:** Classification reports of experimented models.

	**Labels**	**Precision**	**Recall**	**F1-score**	**Support**
SGCNN original	Class 1	0.99	0.99	0.99	355
	Class 2	0.95	0.91	0.93	343
	Class 3	0.68	0.83	0.75	70
	Wei. Avg	0.95	0.94	0.94	768
SGCNN model 1	Class 1	0.99	1.00	0.99	346
	Class 2	0.96	0.93	0.94	342
	Class 3	0.77	0.85	0.81	80
	Wei. Avg	0.95	0.95	0.95	768
SGCNN model 2	Class 1	0.95	0.99	0.97	343
	Class 2	0.95	0.88	0.91	347
	Class 3	0.73	0.85	0.78	78
	Wei. Avg	0.93	0.93	0.93	768
Base CNN model	Class 1	0.99	0.99	0.99	346
	Class 2	0.94	0.89	0.91	341
	Class 3	0.67	0.81	0.73	81
	Wei. Avg	0.93	0.93	0.93	768
Lean CNN model	Class 1	0.98	1.00	0.99	361
	Class 2	0.96	0.90	0.93	335
	Class 3	0.70	0.81	0.75	72
	Wei. Avg	0.94	0.94	0.94	768
Deep CNN model	Class 1	0.99	0.99	0.99	336
	Class 2	0.94	0.93	0.93	351
	Class 3	0.72	0.78	0.75	81
	Wei. Avg	0.94	0.94	0.94	768

The SGCNN Model 1 performs excellently in Class 1, with a precision value of 0.99, a recall value of 1.00, and an F1-score value of 0.99, supported by 346 instances. In Class 2, it achieves a precision value of 0.96, a recall value of 0.93, and an F1-score value of 0.94, supported by 342 instances. Class 3 is handled well, with a precision value of 0.77, a recall value of 0.85, and an F1-score value of 0.81, with support of 80 instances. This model has a weighted average precision, recall, and F1-score of 0.95 for all measures. The SGCNN Model 2 shows robust performance in Class 1, with a precision value of 0.95, a recall value of 0.99, and an F1-score value of 0.97, supported by 343 instances. However, in Class 2, there is a slight drop with a precision value of 0.95, a recall value of 0.88, and an F1-score value of 0.91, with a support of 347 instances. Class 3 is reasonably well handled, with a precision value of 0.73, a recall value of 0.85, and an F1-score value of 0.78, with support of 78 instances. This model has a weighted average precision, recall, and F1-score of 0.93 for all measures. The Base CNN Model demonstrates excellent performance in Class 1, with a precision value of 0.99, a recall value of 0.99, and an F1-score value of 0.99, supported by 346 instances. Class 2 exhibits good performance with a precision value of 0.94, a recall value of 0.89, and an F1-score value of 0.91, supported by 341 instances. The model performs less effectively in Class 3, with a precision value of 0.67, a recall value of 0.81, and an F1-score value of 0.73, supported by 81 instances. This model has a weighted average precision, recall, and F1-score of 0.93 for all measures. The Lean CNN Model maintains strong performance in Class 1, with a precision value of 0.98, a recall value of 1.00, and an F1-score value of 0.99, supported by 361 instances. Class 2 shows a good performance, with a precision value of 0.96, a recall value of 0.90, and an F1-score value of 0.93, with support of 335 instances. The model achieves a precision value of 0.70, a recall value of 0.81, and an F1-score value of 0.75 in Class 3, supported by 72 instances. This model has a weighted average precision, recall, and F1-score of 0.94 for all measures. The Deep CNN Model also shows strong results, particularly in Class 1, with a precision value of 0.99, a recall value of 0.99, and an F1-score value of 0.99, supported by 336 instances. Class 2 maintains good results, with a precision value of 0.94, a recall value of 0.93, and an F1-score value of 0.93, supported by 351 instances. For Class 3, the model achieves a precision value of 0.72, a recall value of 0.78, and an F1-score value of 0.75, supported by 81 instances. The weighted average value of precision, recall, and f1-score is 0.94. Overall, the various CNN models demonstrate strong performance in the frequently observed classes, with some variations in the less frequent Class 3, highlighting different aspects of model robustness and generalization capabilities.

#### 4.2.1 Result of SGCNN original model

**Figure 10:**
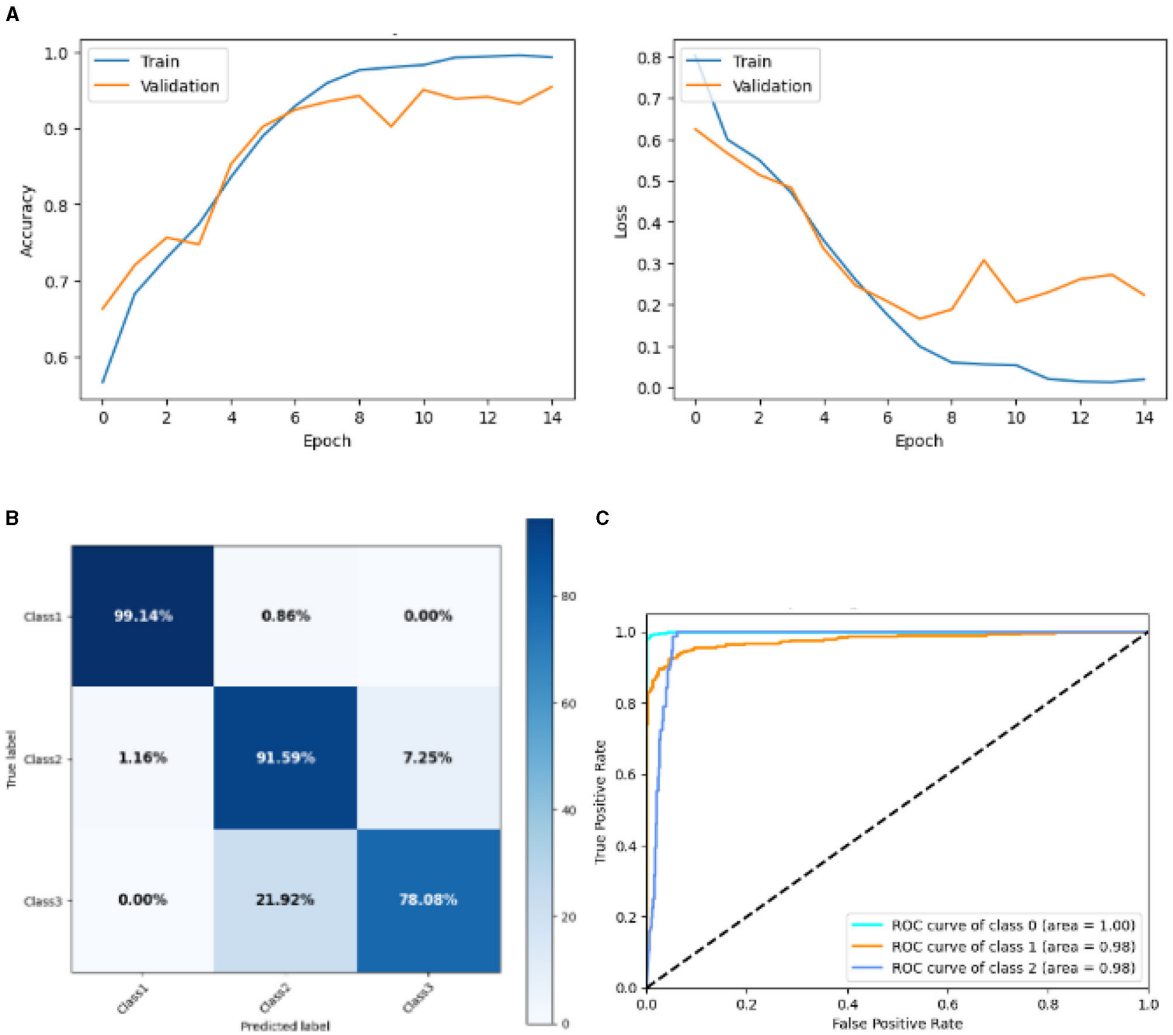
Graphical representation of SGCNN Orginal model's results. **(A)** Accuracy and loss. **(B)** Confusion matrix. **(C)** Receiver operating characteristics curve.

Figure 10 represented the graphical representation of the SGCNN Orginal Model. Figure 10A presents the model loss and accuracy graph of an SGCNN Original Model. The training accuracy starts from 0.50% value at 0*th* epoch, and it increases up to 0.68% at 1*st* epoch. Then, it increases upward, and the training accuracy stops at 1.0% at 14*th* epoch. The validation accuracy starts from 0.67% value at 0*th* epoch. After some fluctuation of increases and decreases, testing accuracy attained is 0.93% at 14*th* epoch. The training loss starts from a 0.8 value at 0*th* epoch, and it decreases downward up to a 0.0 value at 14*th* epoch. The validation loss starts from a 0.6 value at 0*th* epoch and decreases up to 0.2 at 14*th* epoch after going through some fluctuation of increases and decreases. The confusion matrix in Figure 10B gives the percentage of each class that is correctly and incorrectly classified. The elements on the diagonal consist of the classes that have been well-classified, while the non-diagonal elements on the matrix consist of the samples that are misclassified. For class 1, the model correctly classified 99.14% instances and 0.86% of class 1 instances misclassified as class 2. For class 2, the model accurately classified 91.59% instances and 1.16% instances of class 2 incorrectly categorized as class 1 and 7.25% instances of class 2 incorrectly categorized as class 3. For class 3, the model correctly classified 78.08% instances and 21.92% of class 3 instances misclassified as class 2. The performance of the model for each class is shown by the ROC graph in Figure 10C. For different threshold settings, the graph plots the real positive rate against the false positive rate. AUC values for all three classes are very high, as can be seen from the ROC curve, indicating that the model is doing exceptionally well. Class 0 has an AUC of 1.00, class 1 of 0.98, and class 2 of 0.98. For all three classes, this demonstrates the model's extremely high accuracy level. Classes 0, 1, and 3 have cyan, orange, and blue ROC curves, respectively. The model has a low false positive rate and a high true positive rate, as indicated by the fact that all of its ROC curves are located close to the upper left corner. This attests to the model's high classification accuracy among the three classes.

#### 4.2.2 Result of SGCNN model 1

**Figure 11:**
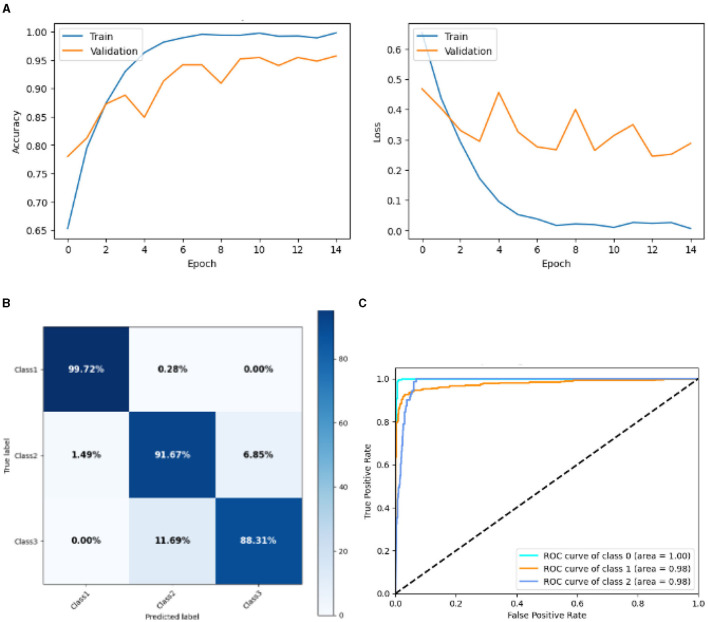
Graphical representation of SGCNN model 1's results. **(A)** Accuracy and loss. **(B)** Confusion matrix. **(C)** Receiver operating characteristics curve.

Figure 11 represented the graphical representation of SGCNN Model 1. Figure 11A presents the model loss and accuracy graph of an SGCNN Model 1. The training accuracy starts from 0.65% value at 0*th* epoch, and it increases up to 0.90% at 1*st* epoch. Then, it increases upward, and the training accuracy stops at 1.00% at 14*th* epoch. The validation accuracy starts from 0.77% value at 0*th* epoch. After some fluctuation of increases and decreases, testing accuracy attained is 0.95% at 14*th* epoch. The training loss starts from a 0.8 value at 0*th* epoch, and it decreases downward up to a 0.0 value at 14*th* epoch. The validation loss starts from a 0.5 value at 0*th* epoch and decreases up to 0.3 value at 14*th* epoch after going through some fluctuation of increases and decreases. The confusion matrix in Figure 11B gives the percentage of each class that is correctly and incorrectly classified. The elements on the diagonal consist of the classes that have been well-classified, while the non-diagonal elements on the matrix consist of the samples that are misclassified. For class 1, the model correctly classified 99.72% instances and 0.28% of class 1 instances misclassified as class 2. For class 2, the model accurately classified 91.67% instances and 1.16% instances of class 2 incorrectly categorized as class 1 and 7.25% instances of class 2 incorrectly categorized as class 3. For class 3, the model correctly classified 78.08% instances and 21.92% of class 3 instances misclassified as class 2. The performance of the model for each class is shown by the ROC graph in Figure 11C. For different threshold settings, the graph plots the real positive rate against the false positive rate. AUC values for all three classes are very high, as can be seen from the ROC curve, indicating that the model is doing exceptionally well. Class 0 has an AUC of 1.00, class 1 of 0.98, and class 2 of 0.98. For all three classes, this demonstrates the model's extremely high accuracy level. Classes 0, 1, and 3 have cyan, orange, and blue ROC curves, respectively. The model has a low false positive rate and a high true positive rate, as indicated by the fact that all of its ROC curves are located close to the upper left corner. This attests to the model's high classification accuracy among the three classes.

#### 4.2.3 Result of SGCNN model 2

**Figure 12:**
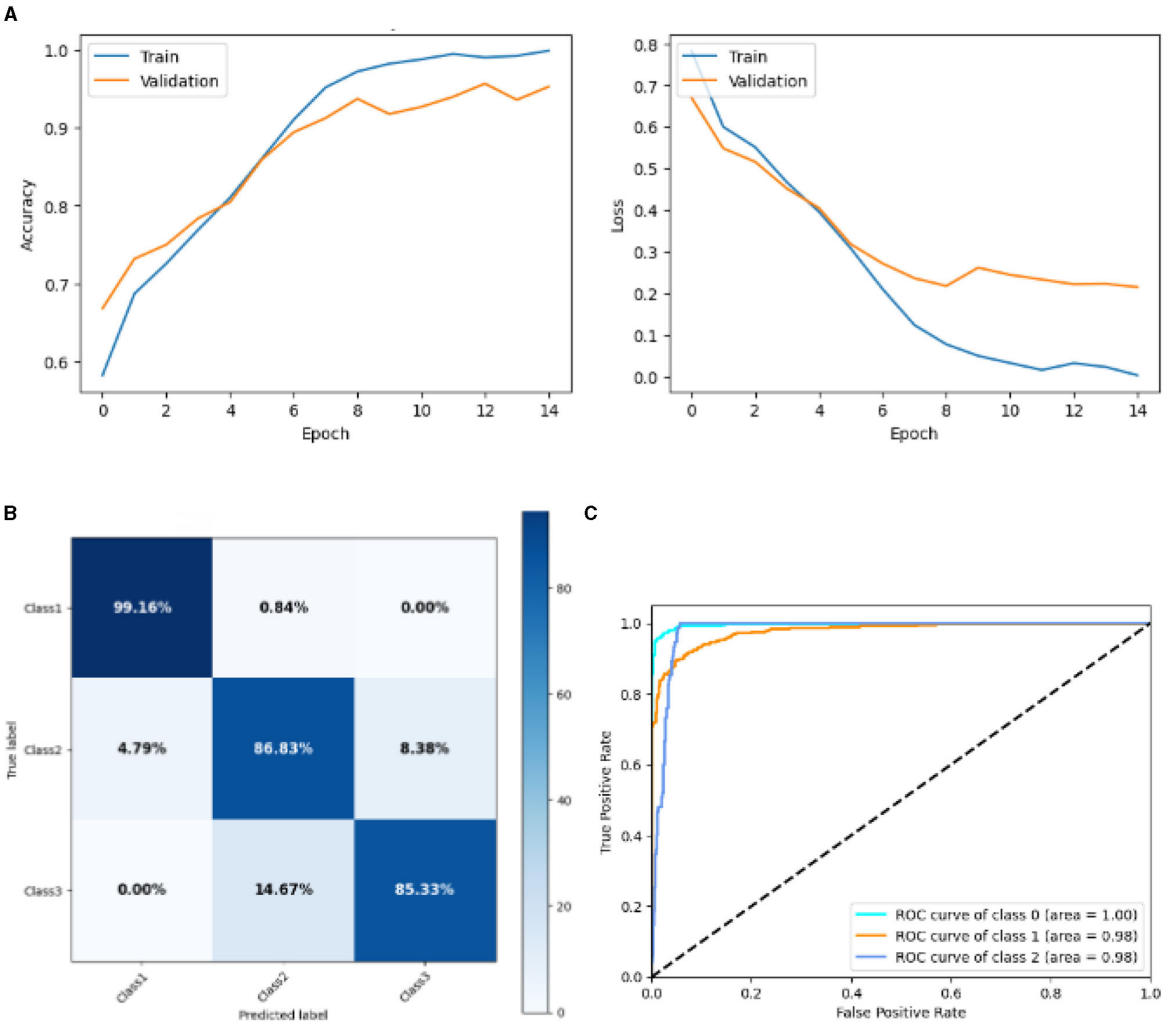
Graphical representation of SGCNN model 2's results. **(A)** Accuracy and loss. **(B)** Confusion matrix. **(C)** Receiver operating characteristics curve.

Figure 12 represented the graphical representation of the SGCNN Orginal model. Figure 12A presents the model's loss and accuracy graph of an SGCNN Model 2. The training accuracy starts from 0.5% value at 0*th* epoch. Then, it increases upward, and the training accuracy stops at 1.00% at 14*th* epoch. The validation accuracy starts from 0.67% value at 0*th* epoch. After some fluctuation of increases and decreases, testing accuracy attained is 0.95% at 14*th* epoch. The training loss starts from a 0.8 value at 0*th* epoch, and it decreases downward up to a 0.0 value at 14*th* epoch. The validation loss starts from a 0.68 value at 0*th* epoch, and it decreases up to a 0.29 value at 14*th* epoch. The confusion matrix in Figure 12B gives the percentage of each class that is correctly and incorrectly classified. The elements on the diagonal consist of the classes that have been well-classified, while the non-diagonal elements on the matrix consist of the samples that are misclassified. For class 1, the model correctly classified 99.16% instances and 0.84% of class 1 instances misclassified as class 2. For class 2, the model accurately classified 86.63% instances and 4.79% instances of class 2 incorrectly categorized as class 1 and 8.38% instances of class 2 incorrectly categorized as class 3. For class 3, the model correctly classified 85.33% instances and 14.67% of class 3 instances misclassified as class 2. The performance of the model for each class is shown by the ROC graph in Figure 12C. For different threshold settings, the graph plots the real positive rate against the false positive rate. AUC values for all three classes are very high, as can be seen from the ROC curve, indicating that the model is doing exceptionally well. Class 0 has an AUC of 1.00, class 1 of 0.98, and class 2 of 0.98. For all three classes, this demonstrates the model's extremely high accuracy level. Classes 0, 1, and 3 have cyan, orange, and blue ROC curves, respectively. The model has a low false positive rate and a high true positive rate, as indicated by the fact that all of its ROC curves are located close to the upper left corner. This attests to the model's high classification accuracy among the three classes.

#### 4.2.4 Result of base CNN model

**Figure 13:**
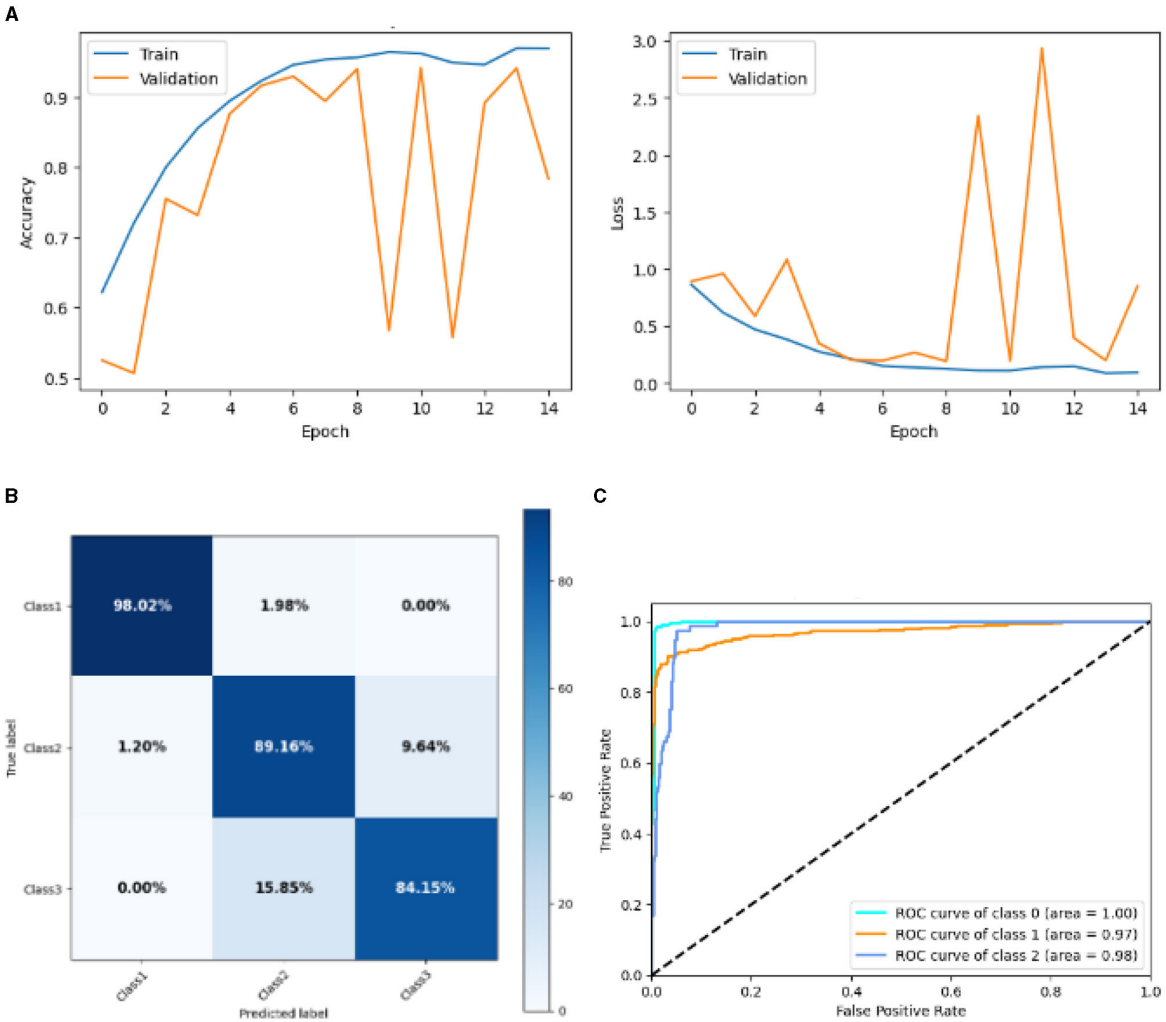
Graphical representation of base CNN model's results. **(A)** Accuracy and loss. **(B)** Confusion matrix. **(C)** Receiver operating characteristics curve.

Figure 13 represented the graphical representation of the Base CNN Model. Figure 13A presents the model accuracy and loss of a Base CNN Model. The training accuracy starts from 0.62% value at 0*th* epoch. Then, it increases upward, and the training accuracy stops at 1.00% at 14*th* epoch. The validation accuracy starts from 0.53% value at 0*th* epoch, and it increases at 0.75% at 2*nd* epoch. After some fluctuation of increases and decreases, validation accuracy attained is 0.79% at 14*th* epoch. The training loss starts from a 0.9 value at 0*th* epoch, and it decreases downward up to a 0.0 value at 14*th* epoch. The validation loss starts from a 0.9 value at 0*th* epoch. It decreases up to 0.6 value at 2*nd*, and then it increases up to 1.1 value at 3*rd* epoch, and then it decreases up to 0.4 value at 4*th* then after going through some fluctuation of increases and decreases the validation loss stops at 0.8 value at 14*th* epoch. The confusion matrix in Figure 13B gives the percentage of each class that is correctly and incorrectly classified. The elements on the diagonal consist of the classes that have been well-classified, while the non-diagonal elements on the matrix consist of the samples that are misclassified. For class 1, the model correctly classified 98.02% instances and 1.98% of class 1 instances misclassified as class 2. For class 2, the model accurately classified 89.16% instances and 1.20% instances of class 2 incorrectly categorized as class 1 and 9.64% instances of class 2 incorrectly categorized as class 3. For class 3, the model correctly classified 84.15% instances and 15.85% of class 3 instances misclassified as class 2. The performance of the model for each class is shown by the ROC graph in Figure 13C. For different threshold settings, the graph plots the real positive rate against the false positive rate. AUC values for all three classes are very high, as can be seen from the ROC curve, indicating that the model is doing exceptionally well. Class 0 has an AUC of 1.00, class 1 of 0.97, and class 2 of 0.98. For all three classes, this demonstrates the model's extremely high accuracy level. Classes 0, 1, and 3 have cyan, orange, and blue ROC curves, respectively. The model has a low false positive rate and a high true positive rate, as indicated by the fact that all of its ROC curves are located close to the upper left corner. This attests to the model's high classification accuracy among the three classes.

#### 4.2.5 Result of lean CNN model

**Figure 14:**
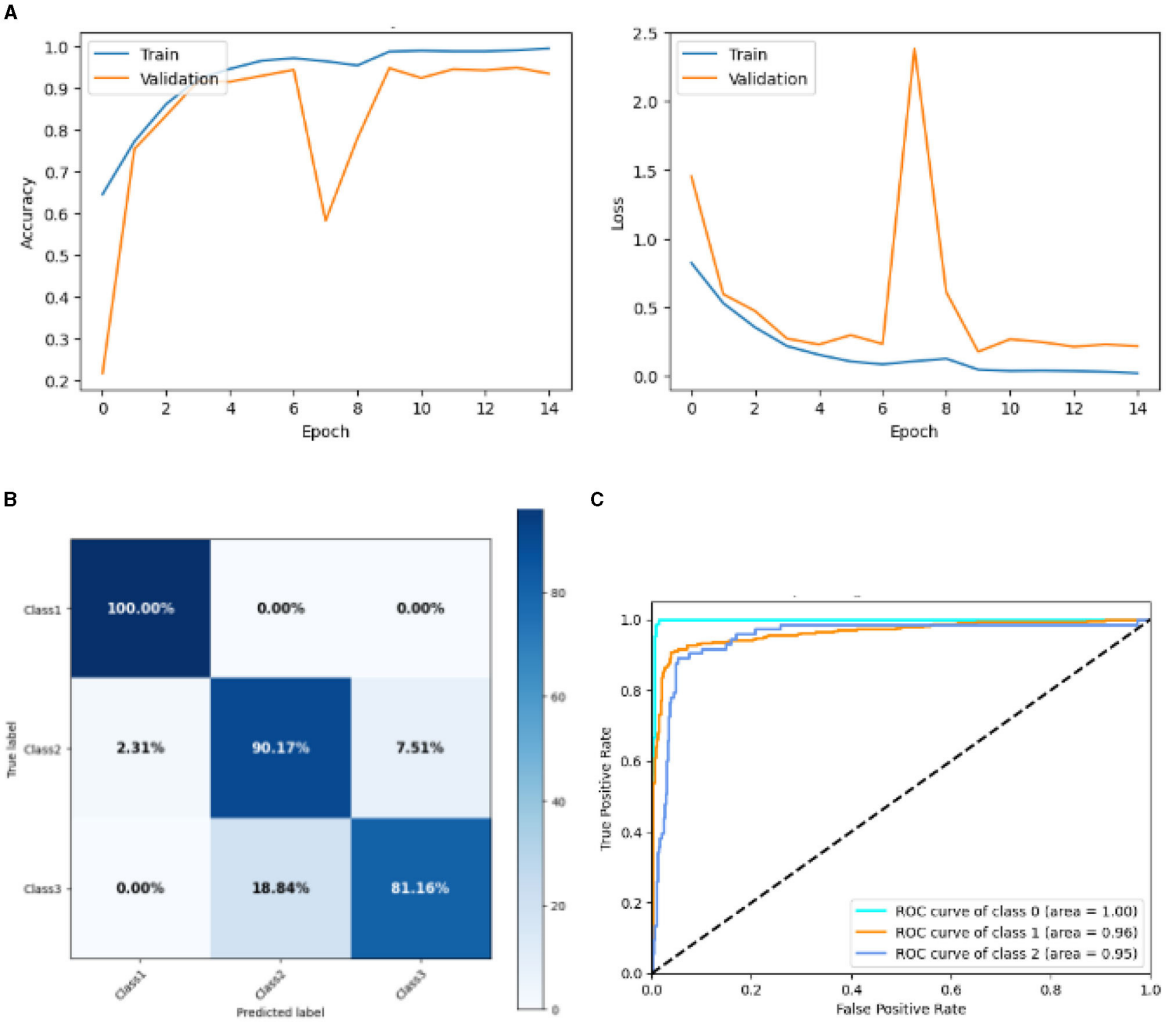
Graphical representation of lean CNN model's results. **(A)** Accuracy and loss. **(B)** Confusion matrix. **(C)** Receiver operating characteristics curve.

Figure 14 represented the graphical representation of the Lean CNN Model. Figure 14A presents the model loss and accuracy of a Lean CNN Model. The training accuracy starts from 0.62% value at 0*th* epoch. Then, it increases upward, and the training accuracy stops at 1.00% at 14*th* epoch. The validation accuracy starts from 0.2% value at 0*th* epoch, and it increases at 0.75% at 1*st* epoch. After some fluctuation of increases and decreases, validation accuracy attained is 0.9% at 14*th* epoch. The training loss starts from a 0.9 value at 0*th* epoch, and it decreases downward up to a 0.0 value at 14*th* epoch. The validation loss starts from a 1.5 value at 0*th* epoch. It decreases up to 0.7 value at 1*st*, and then it increases up to 2.4 value at 7*th* epoch, and then it decreases up to 0.4 value at 14*th* epoch. The confusion matrix in Figure 14B gives the percentage of each class that is correctly and incorrectly classified. The elements on the diagonal consist of the classes that have been well-classified, while the non-diagonal elements on the matrix consist of the samples that are misclassified. For class 1, the model correctly classified 100.00% instances, and 0.00% instances of class 1 were misclassified. For class 2, the model correctly categorized 90.79% instances and 2.31% instances of class 2 misclassified as class 1 and 7.51% instances of class 2 misclassified as class 3. For class 3, the model correctly classified 81.16% instances and 18.84% of class 3 instances misclassified as class 2. Overall, the Lean CNN Model maintains strong performance in Class 1. The performance of the model for each class is shown by the ROC graph in Figure 14C. For different threshold settings, the graph plots the real positive rate against the false positive rate. AUC values for all three classes are very high, as can be seen from the ROC curve, indicating that the model is doing exceptionally well. Class 0 has an AUC of 1.00, class 1 of 0.96, and class 2 of 0.95. For all three classes, this demonstrates the model's extremely high accuracy level. Classes 0, 1, and 3 have cyan, orange, and blue ROC curves, respectively. The model has a low false positive rate and a high true positive rate, as indicated by the fact that all of its ROC curves are located close to the upper left corner. This attests to the model's high classification accuracy among the three classes.

#### 4.2.6 Result of deep CNN model

**Figure 15:**
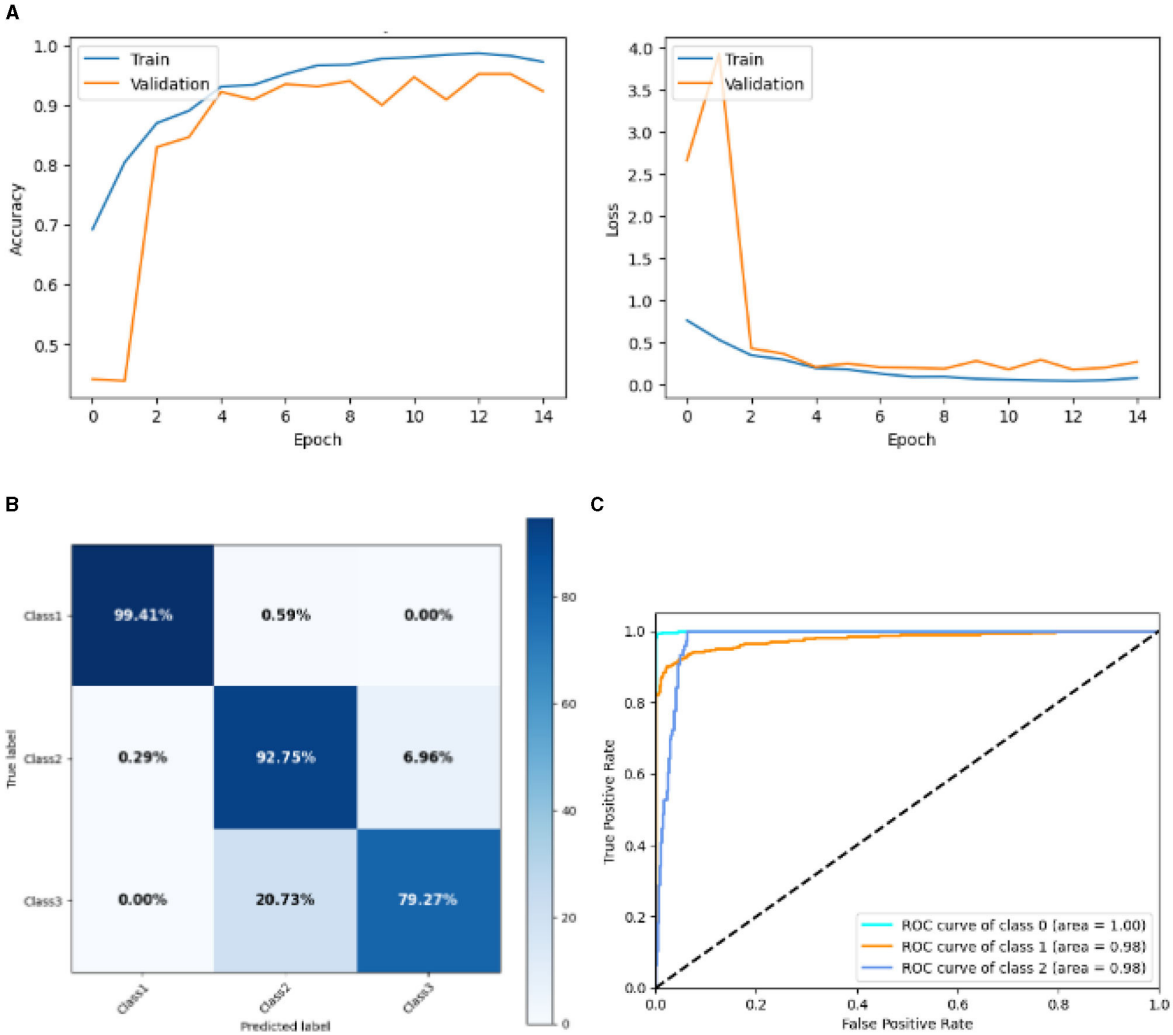
Graphical representation of deep CNN model's results. **(A)** Accuracy and loss. **(B)** Confusion matrix. **(C)** Receiver operating characteristics curve.

Figure 15 represented the graphical representation of the Deep CNN Model. Figure 15A presents the model accuracy and loss of a Deep CNN Model. The training accuracy starts from 0.7% value at 0*th* epoch. Then, it increases upward, and the training accuracy stops at 0.9% at 14*th* epoch. The validation accuracy starts from 0.3% value at 0*th* epoch, and it increases at 0.83% at 2*nd* epoch. After some fluctuation of increases and decreases, validation accuracy attained is 0.9% at 14*th* epoch. The training loss starts from a 0.9 value at 0*th* epoch, and it decreases downward up to a 0.0 value at 14*th* epoch. The validation loss starts from a 2.6 value at 0*th* epoch. It increases up to 4.0 value at 1*st*, and then it decreases up to 0.5 value at 2*nd* epoch, and then some fluctuation of increases and decreases the validation loss stops at 0.4 value at 14*th* epoch. The confusion matrix in Figure 15B gives the percentage of each class that is correctly and incorrectly classified. The elements on the diagonal consist of the classes that have been well-classified, while the non-diagonal elements on the matrix consist of the samples that are misclassified. For class 1, the model correctly classified 99.14% instances, and 0.51% instances of class 1 were misclassified as class 2. For class 2, the model correctly classified 92.75% instances and 0.29% instances of class 2 misclassified as class 1 and 6.96% instances of class 2 misclassified as class 3. For class 3, the model correctly classified 79.27% instances and 20.73% of class 3 instances misclassified as class 2. Overall, the deep CNN Model maintains strong performance in Class 1. The performance of the model for each class is shown by the ROC graph in Figure 15C. For different threshold settings, the graph plots the real positive rate against the false positive rate. AUC values for all three classes are very high, as can be seen from the ROC curve, indicating that the model is doing exceptionally well. Class 0 has an AUC of 1.00, class 1 of 0.98, and class 2 of 0.98. For all three classes, this demonstrates the model's extremely high accuracy level. Classes 0, 1, and 3 have cyan, orange, and blue ROC curves, respectively. The model has a low false positive rate and a high true positive rate, as indicated by the fact that all of its ROC curves are located close to the upper left corner. This attests to the model's high classification accuracy among the three classes.

### 4.3 Discussion and comparison

Table 4 presents a comparative analysis of various models employed for diagnosing and classifying brain-related disorders. The comparison includes studies from different years, showcasing the models used, the datasets involved, and the results achieved. One of the studies from 2022 (Shahwar et al., 2022) employed a Hybrid Classical–Quantum Transfer Learning approach that combined ResNet34 with a Quantum Variational Circuit (QVC). This hybrid model was applied to a dataset focusing on dementia associated with AD. By leveraging the strengths of both classical and quantum computing, the model achieved an accuracy of 92%, demonstrating significant potential for enhancing the machine learning model's performance in dementia detection. In 2023, another study (Nancy Noella and Priyadarshini, 2023) explored multiple classifiers of machine learning, including Multi-class Support Vector Machine (SVM), Naive Bayes, ID3 and Bagged Ensemble. The dataset consisted of PET images representing AD, Parkinson's Disease, and healthy brains. The Bagged Ensemble classifier fared better than the others, obtaining a 90.3% accuracy rate, according to the study. This research demonstrates the precision with which complicated brain disorders can be classified using ensemble learning techniques.

**Table 4:** Comparison of proposed model's result with existing techniques.

**References**	**Year**	**Models**	**Dataset**	**Results**
Shahwar et al. (2022)	2022	Hybrid classical–quantum transfer learning with ResNet34 and Quantum variational circuit (QVC)	Dementia of AD	Accuracy 92%
Nancy Noella and Priyadarshini (2023)	2023	Bagged ensemble, ID3, Naive Bayes, multiclass support vector machine	Image dataset (AD, PD, healthy brain)	Accuracy 90.3%
de Oliveira et al. (2024)	2024	Logistic Regression with L1 and L2 regularization	Images dataset (AD, CN)	AUC 94.75%
Proposed model	**2024**	**SGCNN model 1**	**Image dataset (AD, PD, and CONTROL)**	**Accuracy 95%**

The study (de Oliveira et al., 2024) utilized a logistic regression model with L1 and L2 regularization to diagnose AD. The performance of the model was evaluated using several measures, with the Area Under the Curve (AUC) reaching 94.75%, indicating a strong ability to generalize to unseen neuroimages. The proposed SGCNN model is specifically designed to classify AD images, Parkinson's disease images, and control subjects. This classifier attained an accuracy of 95%, surpassing the performance of previous studies and demonstrating its effectiveness in accurately diagnosing these conditions. The proposed model seems to be effective for AD detection from MRI images because they excel at capturing complex brain connectivity patterns, which are disrupted in AD. MRI data reflects the brain's structural and functional connectivity, which can be represented as a graph, where nodes correspond to brain regions, and edges represent connections between them. Traditional CNNs, designed for grid-like data such as images, struggle with such irregular structures. The proposed model, however, operates on graphs by applying spectral convolutions that capture intricate relationships in the brain's network, enabling them to identify subtle alterations in brain connectivity that are characteristic of Alzheimer's, improving the model's ability to detect the disease accurately. The outcomes demonstrate the elevated precision of the suggested approach and its potential for practical use in the prompt identification and diagnosis of neurodegenerative illnesses.

## 5 Conclusion

The purpose of this study was to increase the diagnostic precision of AD by proposing and evaluating many CNN models for picture categorization. We were able to provide a strong basis for model training and evaluation by carefully splitting our experimental dataset and using strict pre-processing. Further ablation investigations showed that structural alterations could improve performance, as illustrated by the Ablation of SGCNN Model 1, which achieved the maximum accuracy of 95%. The SGCNN Original Model served as a solid baseline with a 93% accuracy. Furthermore, with accuracies ranging from 93% to 94%, the BASE CNN, LEAN CNN, and Deep CNN models showed strong performance. Our results indicate the prospect of the ablation of SGCNN Model 1 as a very powerful tool for classifying AD images, underscoring its potential to support early diagnosis and therapy of AD. Additionally, the constant performance across different models suggests that CNN-based methods can be quite dependable for tasks involving the classification of medical images. To further advance the field of diagnosing neurodegenerative diseases, future studies could focus on enhancing these models by incorporating more diverse and larger datasets, integrating multi-modal data such as genetic or biochemical markers, and exploring real-time applications for early detection and continuous monitoring. Additionally, investigating the use of advanced techniques like transfer learning, ensemble methods, and model interpretability could help improve diagnostic accuracy and reliability in clinical settings.

## Data Availability

The original contributions presented in the study are included in the article/supplementary material, further inquiries can be directed to the corresponding authors.
